# Hypoxia promotes progression of cervical cancer by modulating the ATXN3-enhanced P53 stability or STAT5 phosphorylation

**DOI:** 10.1038/s41420-025-02822-0

**Published:** 2026-01-08

**Authors:** Rong Zhang, Shengjun Chai, Fengjuan Zhang, Jiaming Lai, Rui Wang, Guocai Xu, Xiaoxia Fan, Botong Li, Chunmei Cai

**Affiliations:** 1 Research Center for High Altitude Medicine, Qinghai University Medical College, Xining, Qinghai P. R. China; 2 Key Laboratory of the Ministry of High Altitude Medicine, Qinghai University Medical College, Xining, Qinghai P. R. China; 3 Key Laboratory of Applied Fundamentals of High Altitude Medicine, (Qinghai-Utah Joint Key Laboratory of Plateau Medicine), Qinghai University Medical College, Xining, Qinghai P. R. China; 4 Laboratory for High Altitude Medicine of Qinghai Province, Qinghai University Medical College, Xining, Qinghai P. R. China; 5 Tumor gynaecology Department, The Affiliated Hospital of Qinghai University, Xining, Qinghai P. R. China; 6 Physical Engineering Department, The Fifth People’s Hospital of Qinghai Provincial (Provincial Cancer Hospital), Xining, Qinghai P.R. China

**Keywords:** Cervical cancer, Cell invasion

## Abstract

This study examines hypoxia’s role in regulating ATXN3 (ATXN3) across cervical cancer subtypes and its impact on tumor progression. We analyzed ATXN3 expression in clinical samples and cell lines (C33A, HeLa, SiHa), assessing proliferation/migration/invasion after ATXN3 modulation. The study investigated whether ATXN3 is regulated by hypoxia through hypoxia-inducible factor 1α (HIF-1α). Downstream mechanisms were explored using clinical samples and cell lines, comparing P53 and signal transducer and activator of transcription 5 (STAT5)/p-STAT5 levels between cancer tissues and adjacent non-cancerous tissues, and assessing changes following ATXN3 manipulation. ATXN3 was downregulated in human papillomavirus(HPV18^+^) cervical adenocarcinoma but upregulated in HPV16^+^ cervical squamous cell carcinoma. ATXN3 suppressed malignant behaviors in C33A and HeLa but promoted them in SiHa. HIF-1α expression was elevated in cancer tissues versus non-cancerous tissues, with hypoxic conditions differentially regulating ATXN3 via HIF-1α across cell lines. Cervical cancer tissues showed lower P53 and higher p-STAT5 (in HPV16+ squamous cell carcinoma). ATXN3 overexpression stabilized P53 in C33A/HeLa and increased p-STAT5 in SiHa, with inverse effects upon silencing. The findings suggest that hypoxia promotes the progression of subtypes of cervical cancer by regulating ATXN3-enhanced P53/p-STAT5 levels, which may provide a novel therapeutic strategy for clinical applications.

## Introduction

Cervical cancer remains a major global health issue, causing approximately 661,000 new cases and 348,000 deaths annually, with a disproportionate impact in low-income regions where screening is limited [1, 2]. Although Human papillomavirus (HPV) vaccination has reduced high-grade cervical lesions by 31–51% in vaccinated groups, the absolute number of cases rose by 62.9% between 1990 and 2021 [3, 4]. Treatment is challenged by chemoresistance mediated by non-coding RNAs, limited access to costly targeted therapies such as bevacizumab, emerging resistance to newer drugs, and incomplete validation of immunotherapies [5–10]. Disease heterogeneity further complicates management: squamous cell carcinoma (SCC, 75%) and adenocarcinoma (AC, 20%) differ substantially, with AC showing consistently worse survival outcomes, while HPV^-^ cases are rarer and more aggressive [11–15]. A deeper understanding of subtype-specific pathogenic mechanisms is essential to identify novel therapeutic targets and improve cervical cancer treatment.

Deubiquitinases, comprising over 100 members across seven subfamilies, play crucial yet context-dependent roles in cervical cancer [16]. Some, such as ubiquitin-specific protease 26 (USP26) and cylindromatosis, function as tumor suppressors [17, 18], while others including USP13, OTU deubiquitinase 1, and USP18 promote oncogenesis [19–21]. Within the Josephin domain-containing Deubiquitinases, ataxin-3 (ATXN3) is the most well-characterized and exhibits tissue-specific duality: it acts as an oncogene in breast and prostate cancers but as a tumor suppressor in gastric and colorectal cancers[22–26] This functional ambiguity underscores the importance of investigating its role in cervical cancer subtypes. Mechanistically, ATXN3 can promote apoptosis by deubiquitinating and stabilizing tumor protein P53 (P53), a well-established suppressor of cervical cancer progression [27–30]. However, the regulatory relationship between ATXN3 and P53 in cervical cancer remains poorly understood, highlighting its potential as a promising therapeutic target that warrants further mechanistic exploration in cervical cancer biology.

Solid tumors often develop hypoxic regions that drive progression and therapy resistance in cervical cancer [31]. Under low oxygen, hypoxia-inducible factor-1α (HIF-1α) stabilizes and acts as a key transcriptional regulator, altering the expression of genes involved in metabolism, epigenetics, and post-translational modification [32, 33]. Although HIF-1α is known to influence deubiquitinases, its role in regulating ATXN3 in cervical cancer was previously unclear [34]. In this study, we found that ATXN3 is differentially expressed across subtypes and has opposing functions: In both in vivo and in vitro experiments, it suppresses malignant behavior in C33A (HPV⁻ SCC) and HeLa (HPV16^+^ AC) cells but promotes it in SiHa (HPV18^+^ SCC) cells. Hypoxia differentially modulates ATXN3 via HIF-1α—downregulating it in C33A and HeLa cells, while upregulating it in SiHa cells. Mechanistically, ATXN3 stabilizes P53 in C33A and HeLa models, inhibiting tumor growth, whereas in SiHa it enhances STAT5 phosphorylation, supporting progression. These findings establish ATXN3 as a context-dependent effector with subtype-specific regulatory mechanisms, highlighting its potential as a therapeutic target in cervical cancer.

## Results

### ATXN3 expressed differentially between subtypes of human cervical cancer tissues

**Fig. 1:**
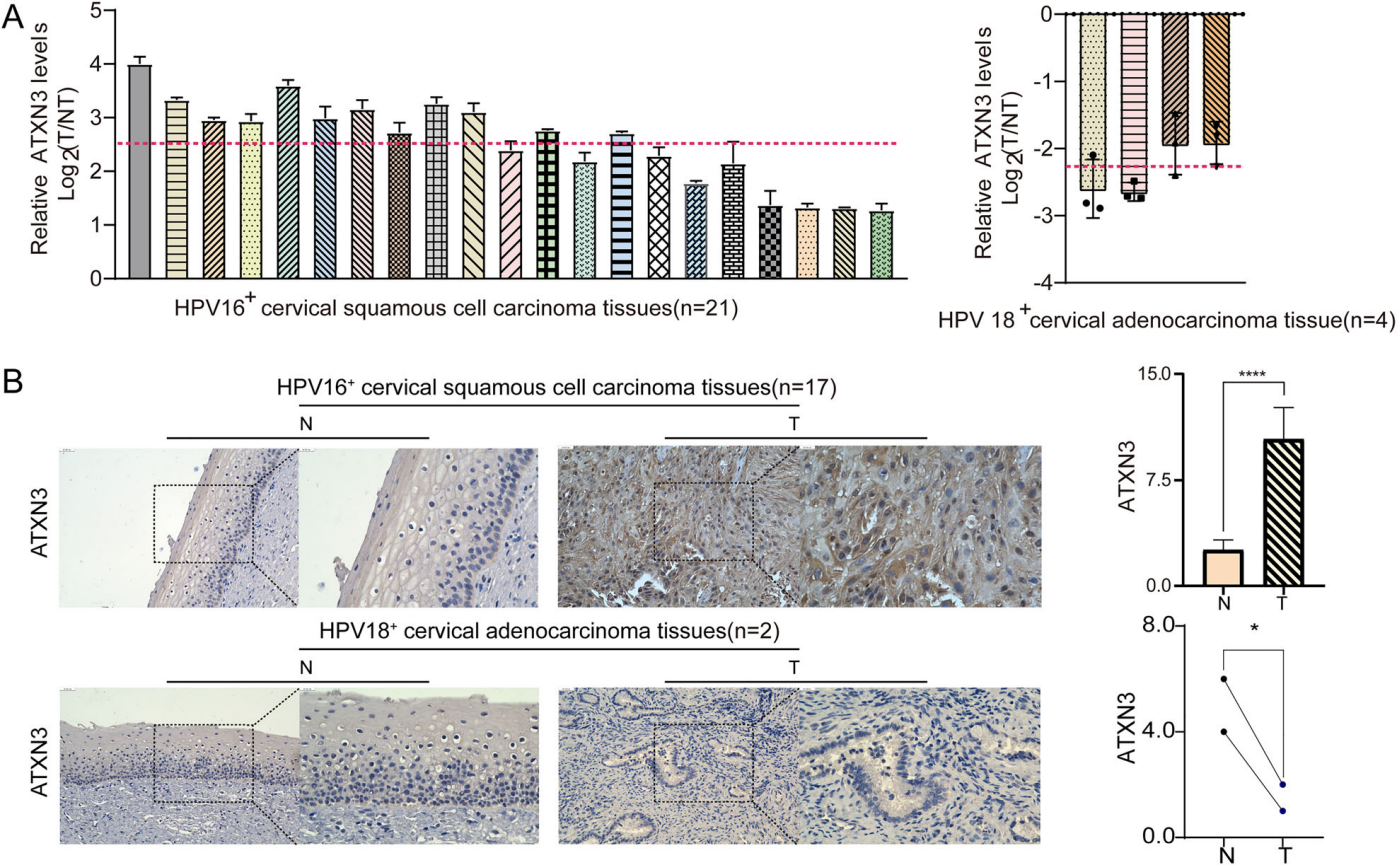
Expression of ATXN3 in human cervical cancer tissue subtypes. **A** ATXN3 mRNA expression levels in different human cervical cancer tissue subtypes. Tumor tissues analyzed: 21 HPV16^+^ SCCs (left) and 4 HPV18^+^ ACs (right). The qPCR data were analyzed using the comparative ΔΔCq method. The Cq values of the target gene were normalized to the endogenous control gene to calculate ΔCq values. For each matched pair of tumor (T) and adjacent non-tumor (NT) tissues, the ΔΔCq value was calculated. The relative gene expression change was expressed as log₂(fold change) = -ΔΔCq. The log₂(fold change) values from all samples were compared to the hypothetical value of 0 using a one-sample *t* test to determine statistical significance. The data are presented as the log₂(fold change) for individual samples. **B** Differential expression of ATXN3 protein in different human cervical cancer tissue subtypes. Tumor tissues analyzed: 17 HPV16^+^ SCCs (top) and 2 HPV18^+^ ACs (bottom). The statistical notations used in the figure are:ns *P* > 0.05, **P < 0.01, ***P < 0.001, and ****P < 0.0001. Data are represented as mean ± SD. For comparisons involving the HPV18^+^ AC group (*n* = 2 or 4), the Mann–Whitney U test was used. All other comparisons between two groups were performed using a two-tailed unpaired Student’s *t* test.

To elucidate the functional role of ATXN3 in cervical carcinogenesis, we analyzed its expression in tumor tissues and matched adjacent non-tumor samples. RT-qPCR revealed subtype-specific differential expression: ATXN3 mRNA was upregulated approximately 6-fold in HPV16^+^ SCC tissues, but downregulated around 5-fold in HPV18^+^ AC tissues compared to normal controls (Fig. 1A). IHC confirmed these patterns at the protein level, with strong ATXN3 staining in HPV16^+^ SCC and weak staining in HPV18^+^ AC specimens (Fig. 1B). These consistent, opposing expression profiles suggest distinct subtype-specific roles for ATXN3 in cervical cancer development.

### Hypoxia regulates ATXN3 levels via HIF-1α in different cervical cancer subtypes to promote tumor progression

**Fig. 2:**
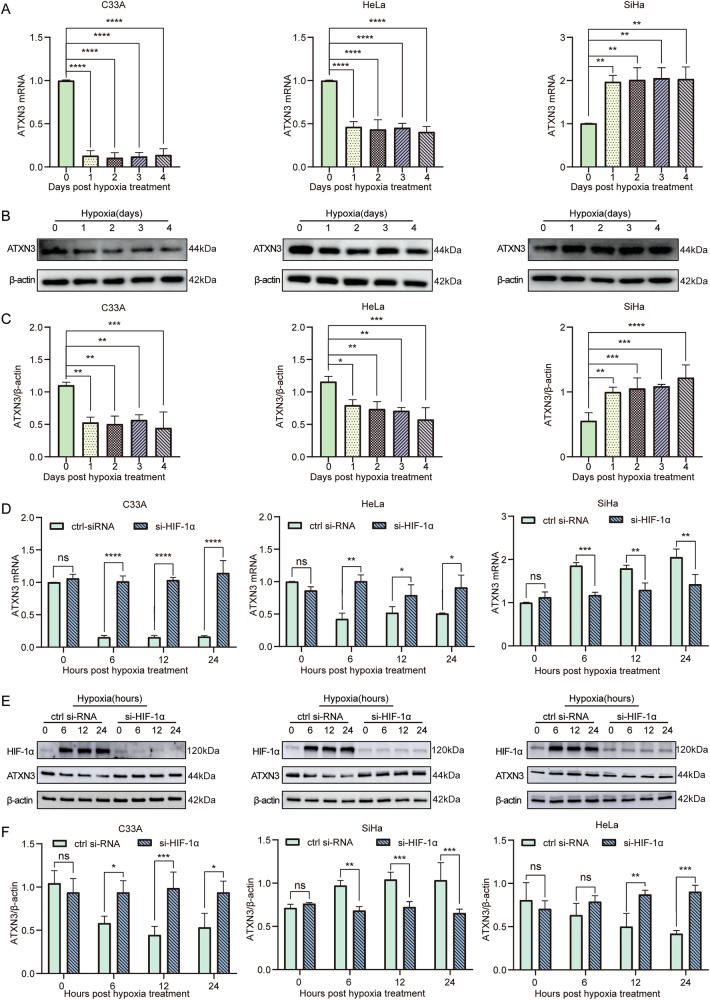
Effects of hypoxia and HIF-1α on ATXN3 in cervical cancer cell lines. **A** Impact of hypoxia on ATXN3 mRNA expression in C33A, HeLa, and SiHa cells. Each individual data point represents an independent cell culture experiment. **B** Influence of hypoxia on ATXN3 protein expression levels in C33A, HeLa, and SiHa cells. (n ≥ 3 independent cell cultures). **C** Statistical analysis of hypoxia-induced changes in ATXN3 protein expression in C33A, HeLa, and SiHa cells. Each individual data point represents an independent cell culture experiment. **D** Alterations in ATXN3 mRNA levels following HIF-1α knockdown under hypoxic conditions in C33A, HeLa, and SiHa cells. Each individual data point represents an independent cell culture experiment. **E** Effect of HIF-1α siRNA transfection on ATXN3 protein expression in hypoxic C33A, HeLa, and SiHa cells. (n ≥ 3 independent cell cultures). **F** Statistical analysis of HIF-1α siRNA-mediated changes in ATXN3 protein expression under hypoxia in C33A, HeLa, and SiHa cells. Each individual data point represents an independent cell culture experiment. The statistical notations used in the figure are:ns *P* > 0.05, **P < 0.01, ***P < 0.001, and ****P < 0.0001. Data are presented as mean ± SD. One-way ANOVA was employed for analyses in panels (**A** and **C**), while multiple comparisons using two-way ANOVA were performed for panels (**D** and **F**).

Cell-based experiments confirmed that hypoxia promotes proliferation, migration, and invasion in cervical cancer cells (Fig. S1A–D). To determine whether hypoxia influences ATXN3 expression—previously shown to be subtype-specific in clinical samples—we examined three representative cell lines: HeLa (HPV18^+^ AC), SiHa (HPV16^+^ SCC), and C33A (HPV⁻SCC). Under both short- and long-term hypoxia, ATXN3 mRNA and protein levels were downregulated in C33A and HeLa cells but upregulated in SiHa cells (Figs. S2A–C, 2A–C). These changes occurred rapidly (within 6 h) and were more pronounced at the mRNA level, especially in C33 A cells. To further validate whether hypoxia regulates ATXN3 through HIF-1α. in cervical cancer. First, we successfully validated the knockdown efficiency of HIF-1α protein levels under hypoxic conditions across all three cell lines (Fig. S2D). Later investigation revealed that HIF-1α knockdown under hypoxia increased ATXN3 expression in C33A and HeLa cells while decreasing it in SiHa cells, effects observed at both mRNA and protein levels (Fig. 2D–F). To identify potential binding targets of HIF-1α on ATXN3, we predicted the hypoxia response elements (HREs) of HIF-1α (Table S1) and identified multiple putative binding sites (Table S2-3), suggesting direct transcriptional regulation. These results establish ATXN3 as a downstream target of the hypoxia–HIF-1α axis and highlight its context-dependent role across cervical cancer subtypes.

### ATXN3 upregulated inhibited proliferation, migration and invasion of C33A and HeLa, while SiHa showed the opposite

**Fig. 3:**
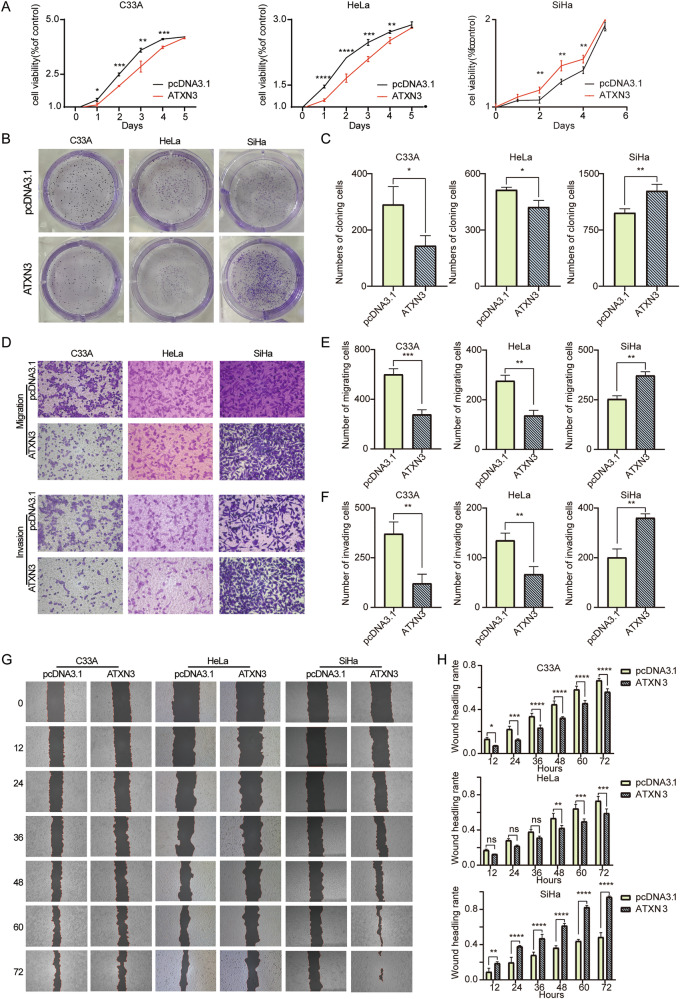
Effects of ATXN3 upregulation on proliferation, migration and invasion of cervical cancer cells. **A** MTS assay performed in C33A, HeLa and SiHa cells following ATXN3 overexpression. (*n* = 3 independent cell cultures) **B** Impact of ATXN3 overexpression on colony formation numbers in C33A, HeLa and SiHa cells. (*n* = 3 independent cell cultures). **C** Statistical analysis of ATXN3 overexpression effects on colony formation numbers in C33A, HeLa and SiHa cells. Each individual data point represents an independent cell culture experiment. **D** Transwell assay evaluating migration and invasion capacities of C33A, HeLa and SiHa cells with ATXN3 overexpression. (*n* = 3 independent cell cultures). **E** Statistical analysis of Transwell migration assay results for ATXN3-overexpressing C33A, HeLa and SiHa cells. Each individual data point represents an independent cell culture experiment. **F** Statistical analysis of Transwell invasion assay results for ATXN3-overexpressing C33A, HeLa and SiHa cells. Each individual data point represents an independent cell culture experiment. **G** Wound healing assay assessing the effect of ATXN3 upregulation on migration capacity of C33A, HeLa and SiHa cells. (*n* = 3 independent cell cultures). **H** Statistical analysis of wound healing assay results demonstrating ATXN3 upregulation effects on migration of C33A, HeLa and SiHa cells. Each individual data point represents an independent cell culture experiment. The statistical notations used in the figure are:ns *P* > 0.05, **P < 0.01, ***P < 0.001, and ****P < 0.0001. Data are represented as mean ± SD. Two-tailed unpaired Student’s *t* test was employed for analyses in panels (**C**, **E**, and **F**), while multiple comparisons using two-way ANOVA were performed for panels (**A** and **H**).

To investigate the functional role of ATXN3 in cervical cancer, we successfully overexpressed ATXN3 in C33A, HeLa, and SiHa cells, with HeLa exhibiting the highest induction—approximately fivefold greater than the other lines (Fig. S3A, B). MTS assays revealed that ATXN3 overexpression suppressed proliferation in C33A and HeLa cells but enhanced it in SiHa cells, with the most significant effects occurring between days 2–4 (P < 0.0001; Fig. 3A). Colony formation assays further confirmed these subtype-specific effects: ATXN3 upregulation reduced colony numbers in C33A and HeLa cells—most markedly in C33A, with a nearly 50% decrease—while increasing colony formation in SiHa cells (Fig. 3B, C). Transwell and wound healing assays consistently showed that ATXN3 overexpression inhibited migration and invasion in C33A and HeLa cells but strongly promoted both processes in SiHa cells (Fig. 3D–F). Wound closure was significantly delayed in C33A and HeLa cells, whereas SiHa exhibited nearly complete healing within 72 h (Fig. 3G, H). The inhibitory effect in HeLa became statistically significant only at 48 h, indicating a comparatively milder impact. Collectively, these results demonstrate that ATXN3 exerts opposing, context-dependent roles across cervical cancer subtypes, suppressing malignant phenotypes in C33A and HeLa while promoting them in SiHa cells.

### Knockdown of ATXN3 increased the proliferation, migration and invasion of C33A and HeLa, but it suppressed SiHa cells

**Fig. 4:**
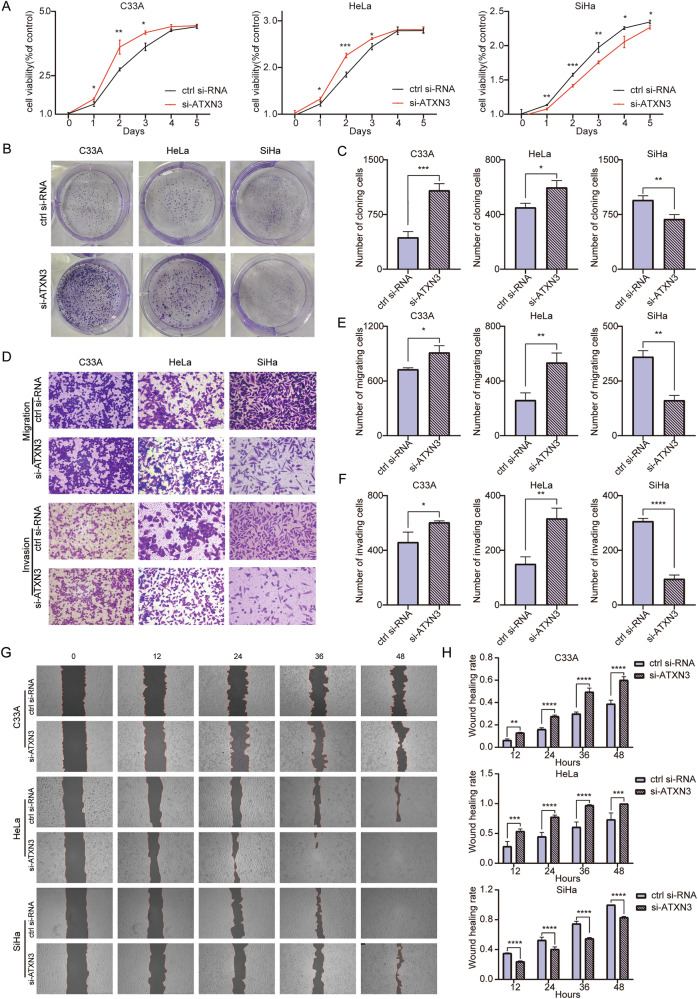
Effects of ATXN3 knockdown on proliferation, migration, and invasion of cervical cancer cells. **A** MTS assay assessing the impact of ATXN3 knockdown on viability in C33A, HeLa, and SiHa cells. (*n* = 3 independent cell cultures). **B** Colony formation assay evaluating proliferative capacity changes in C33A, HeLa, and SiHa cells following ATXN3 downregulation. (*n* = 3 independent cell cultures). **C** Statistical analysis of ATXN3 knockdown effects on colony formation numbers in C33A, HeLa, and SiHa cells. Each individual data point represents an independent cell culture experiment. **D** Transwell migration and invasion assays performed after ATXN3 silencing in C33A, HeLa, and SiHa cells. (*n* = 3 independent cell cultures). **E** Statistical analysis of transwell migration assay results for ATXN3-silenced C33A, HeLa, and SiHa cells. Each individual data point represents an independent cell culture experiment. **F** Statistical analysis of Transwell invasion assay results for ATXN3-silenced C33A, HeLa, and SiHa cells. Each individual data point represents an independent cell culture experiment. **G** Wound healing assay measuring migration capacity of C33A, HeLa, and SiHa cells upon ATXN3 silencing. (*n* = 3 independent cell cultures). **H** Statistical analysis of wound healing assay results demonstrating ATXN3 downregulation effects on migration in C33A, HeLa, and SiHa cells. Each individual data point represents an independent cell culture experiment. The statistical notations used in the figure are:ns *P* > 0.05, **P < 0.01, ***P < 0.001, and ****P < 0.0001. Data are represented as mean ± SD. Two-tailed unpaired Student’s *t* test was employed for analyses in panels (**C**, **E** and **F**), while multiple comparisons using two-way ANOVA were performed for panels (**A** and **H**).

Following ATXN3 siRNA transfection, mRNA and protein levels were successfully reduced in all three cervical cancer cell lines, with the most pronounced decrease observed in HeLa cells (Fig. S3C, D). Knockdown of ATXN3 enhanced proliferation, colony formation, migration, and invasion in C33A and HeLa cells, but suppressed these processes in SiHa cells (Fig. 4A–H). Significant changes in proliferation were detected as early as day 1 post-knockdown, with the strongest effects on day 2. Colony formation increased over two-fold in C33A cells (*P* < 0.001), while more moderate changes occurred in HeLa and SiHa cells. Transwell and wound healing assays confirmed that ATXN3 depletion strongly promoted migration and invasion in C33A and HeLa cells—with HeLa achieving complete wound closure within 48 h—while significantly inhibiting these capabilities in SiHa cells (*P* < 0.01). These results, consistent with overexpression data, demonstrate that ATXN3 knockdown exerts subtype-specific and functionally opposing effects on cervical cancer cell behavior.

### Rescuing the expression of ATXN3 abolishes the hypoxia-induced proliferation, migration, and invasion of cervical cancer cells

**Fig. 5:**
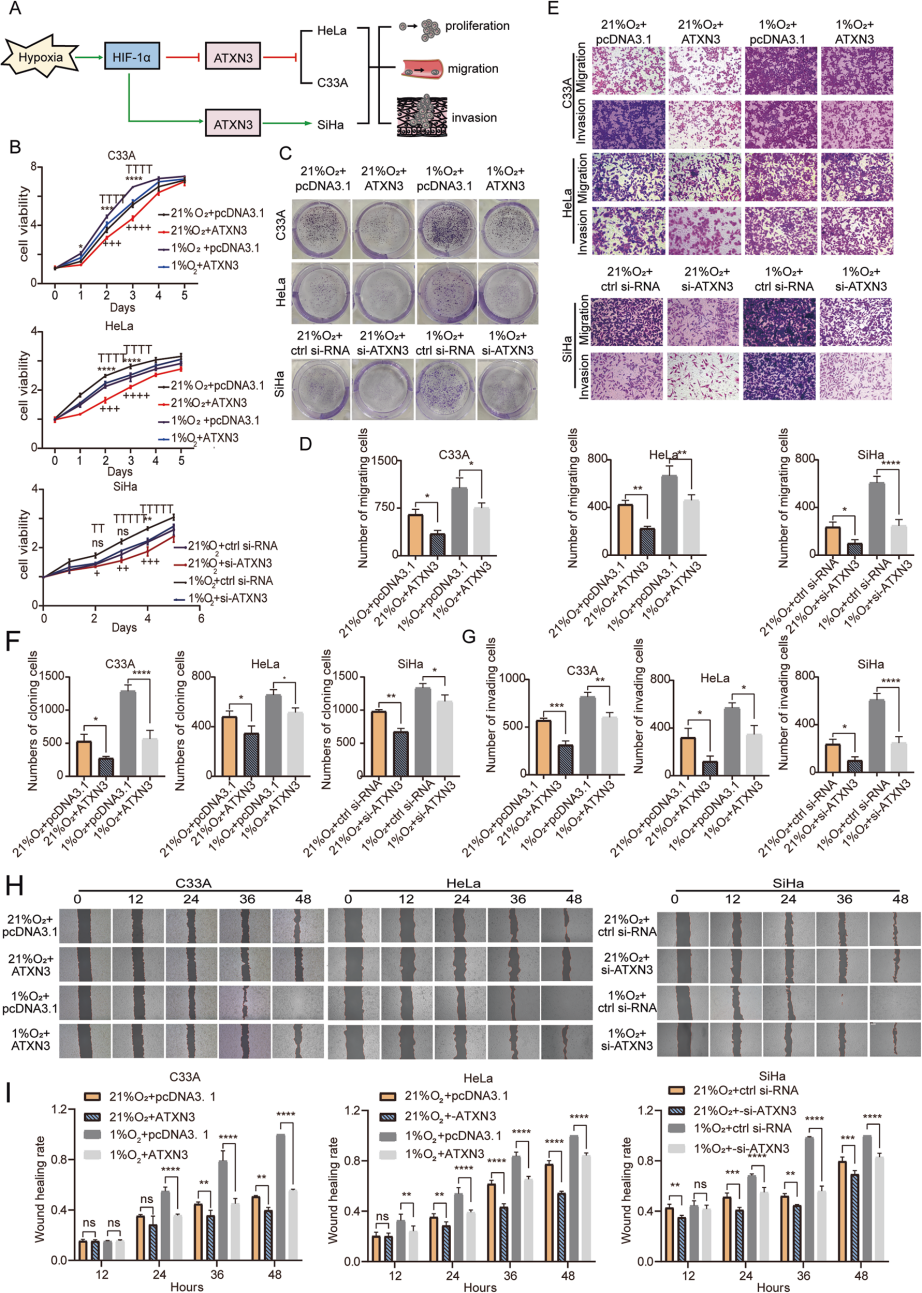
Effects of ATXN3 rescue on proliferation, migration, and invasion capacities of cervical cancer cells following hypoxia treatment. **A** Schematic illustrating hypoxia-mediated regulation of ATXN3 levels through HIF-1α to promote proliferation, migration, and invasion across cervical cancer subtypes. **B** MTS assay evaluating proliferative capacity under hypoxia after ATXN3 overexpression in C33A/HeLa cells or ATXN3 knockdown in SiHa cells. (*n* = 3 independent cell cultures). **C** Colony formation assay assessing colony numbers post-ATXN3 manipulation under hypoxic conditions. (*n* = 3 independent cell cultures). **D** Statistical analysis of hypoxia-induced colony formation changes following ATXN3 rescue. Each individual data point represents an independent cell culture experiment. **E** Transwell assays demonstrating migration/invasion capacities after ATXN3 modulation during hypoxia. (*n* = 3 independent cell cultures). Quantitative analyses of transwell migration (**F**) and invasion (**G**) results under combinatorial hypoxia/ATXN3 treatments. Each individual data point represents an independent cell culture experiment. **H** Wound healing assays showing migratory recovery after ATXN3 rescue in hypoxic conditions. (*n* = 3 independent cell cultures). **I** Statistical validation of wound closure rates following combined hypoxia exposure and ATXN3 manipulation. Each individual data point represents an independent cell culture experiment. The statistical notations used in the figure are:ns *P* > 0.05, **P < 0.01, ***P < 0.001, and ****P < 0.0001 comparing the 1% O2 + pcDNA3.1/ctrl si-RNA group with the 1% O2 + ATXN3/si-ATXN3 group; TTP < 0.01, TTTTP < 0.0001 comparing the 21% O2 + pcDNA3.1/ctrl si-RNA group with the 1% O2 + pcDNA3.1/ctrl si-RNA group; +*P* < 0.05, ++*P* < 0.01, +++*P* < 0.001, and ++++*P* < 0.0001 comparing the 21% O2+ pcDNA3.1/ctrl si-RNA group with the 21% O2 + ATXN3/si-ATXN3 group. Data are represented as mean ± SD. One-way ANOVA was employed for analyses in panels (**D**, **F** and **G**), while multiple comparisons using two-way ANOVA were performed for panels (**B** and **I**).

In summary, hypoxia was shown to downregulate ATXN3 via HIF-1α in C33A and HeLa cells, promoting proliferation, migration, and invasion, while upregulating ATXN3 in SiHa cells and similarly facilitating malignant progression (Fig. 5A). To determine whether these hypoxia-induced phenotypes were ATXN3-dependent, we performed genetic rescue experiments: ATXN3 was overexpressed in hypoxic C33A and HeLa cells and knocked down in hypoxic SiHa cells (Fig. S4A, B). MTS and colony formation assays demonstrated that ATXN3 overexpression reversed hypoxia-enhanced proliferation in C33A and HeLa, whereas ATXN3 knockdown suppressed it in SiHa (Fig. 5B, C). The effect was most pronounced in C33A cells (*P* < 0.0001). Similarly, Transwell and wound healing assays confirmed that ATXN3 modulation counteracted hypoxia-driven migration and invasion: overexpression reduced migration and invasion by approximately 50% in C33A and HeLa (*P* < 0.05), while knockdown led to a 67% reduction in SiHa (*P* < 0.0001) (Fig. 5E–G). Wound closure was significantly delayed in all three cell types upon ATXN3 reversal, with the strongest effect observed in C33A as early as 24 h (*P* < 0.0001; Fig. 5H, I). These findings establish ATXN3 as a central mediator of hypoxia-induced malignancy in a subtype-specific manner, and demonstrate that targeted manipulation of ATXN3 expression can reverse hypoxic effects across cervical cancer subtypes.

### ATXN3 knockdown exerts subtype-specific effects on cervical cancer tumor growth in vivo

**Fig. 6:**
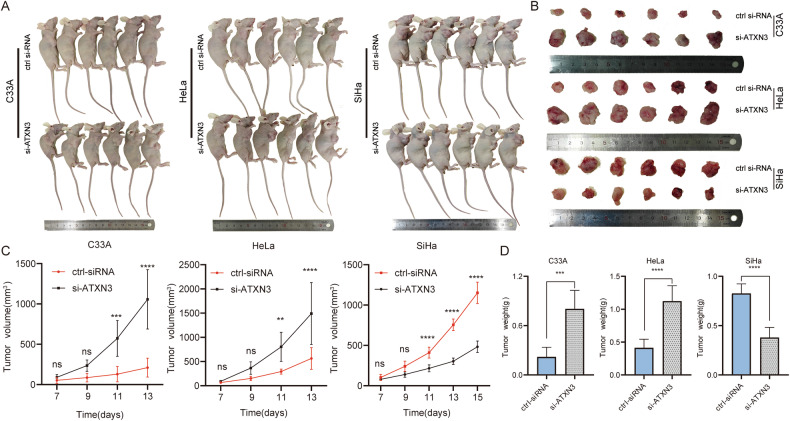
In vivo experiments on the effects of ATXN3 silencing in cervical cancer subtypes. **A**, **B** Effect of ATXN3 knockdown on tumor size across different subtypes (*n* = 6). **C** Impact of ATXN3 knockdown on xenograft volume (*n* = 6). **D** Effect of ATXN3 knockdown on xenograft weight (*n* = 6). The statistical notations used in the figure are:ns *P* > 0.05, **P < 0.01, ***P < 0.001, and ****P < 0.0001. Data are represented as mean ± SD. multiple comparisons using two-way ANOVA were performed for panel C, while two-tailed unpaired Student’s *t* test was employed for analyses in panel (**D**).

To evaluate the role of ATXN3 in vivo, we first validated its knockdown efficiency in C33A, HeLa, and SiHa cells (Fig. S5A–C), then established xenograft models using these silenced cells. ATXN3 knockdown significantly promoted tumor growth in C33A and HeLa models, while strongly suppressing it in SiHa-bearing mice (Fig. 6A, B). Statistically significant differences in tumor growth emerged by day 11 across all groups (*P* < 0.05). By day 13, C33A tumors with ATXN3 knockdown showed approximately fivefold increases in volume and weight compared to controls, and HeLa tumors exhibited threefold enhancements. In contrast, SiHa tumors with ATXN3 knockdown were reduced to roughly one-third the size and weight of controls by day 15 (Fig. 6C, D). These in vivo results confirm the subtype-specific role of ATXN3, consistent with our in vitro findings, demonstrating that ATXN3 silencing enhances tumor growth in C33A and HeLa contexts but inhibits it in the SiHa model.

### ATXN3 modulates different subtypes of cervical cancer tissues by either stabilizing P53 or enhancing STAT5 phosphorylation

**Fig. 7:**
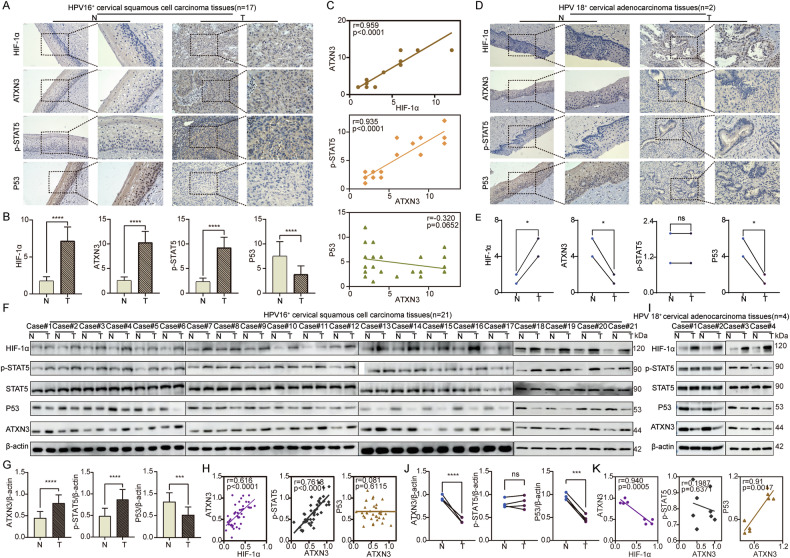
ATXN3 promotes the progression of different subtypes of cervical cancer tissues through P53 or p-STAT5. **A** IHC detection of HIF1-α, ATXN3, P53, and p-STAT5 proteins in HPV16^+^ cervical SCC tissues. (*n* = 17 HPV16^+^ SCCs). **B** Statistical analysis of protein levels in HPV16^+^ cervical SCC tissues by IHC. (*n* = 17 HPV16^+^ SCCs). **C** Correlation analysis of protein levels in HPV16^+^ cervical SCC tissues by IHC. (n = 17 HPV16^+^ SCCs). **D** IHC detection of HIF1-α, ATXN3, P53, and p-STAT5 proteins in HPV18^+^ cervical AC tissues. (*n* = 2 HPV18^+^ ACs). **E** Statistical analysis of protein levels in HPV18^+^ cervical AC tissues by IHC. (*n* = 2 HPV18^+^ ACs). **F** WB assay detecting the protein levels of HIF1-α, ATXN3, P53, and p-STAT5 in HPV16^+^ cervical SCC tissues and adjacent normal tissues. (*n* = 21 HPV16^+^ SCCs). **G** Statistical analysis of ATXN3, P53, and p-STAT5 protein levels in HPV16^+^ cervical SCC tissues and adjacent normal tissues by WB. (*n* = 21 HPV16^+^ SCCs). **H** Correlation analysis of protein levels in HPV16^+^ cervical SCC tissues and adjacent normal tissues by WB. (*n* = 21 HPV16^+^ SCCs) **I** WB assay detecting the protein levels of HIF1-α, ATXN3, P53, and p-STAT5 in HPV18^+^ cervical AC tissues and adjacent normal tissues. (*n* = 4 HPV18^+^ ACs). **J** Statistical analysis of ATXN3, P53, and p-STAT5 protein levels in HPV18^+^ cervical AC tissues and adjacent normal tissues by WB. (*n* = 4 HPV18^+^ ACs). **K** Correlation analysis of protein levels in HPV18^+ ^AC tissues and adjacent normal tissues by WB. (*n* = 4 HPV18^+^ ACs) The statistical notations used in the figure are:ns *P* > 0.05, **P < 0.01, ***P < 0.001, and ****P < 0.0001. Data are represented as mean ± SD. For comparisons involving the HPV18^+^ AC group (*n* = 2 or 4), the Mann-Whitney U test was used. All other comparisons between two groups were performed using a two-tailed unpaired Student’s *t* test, while linear regression analysis were performed for panels (**C** and **H**).

Our study reveals that ATXN3 exhibits dual, context-dependent roles in cervical cancer—acting as either a tumor suppressor or an oncoprotein depending on histological and HPV status. Gene Set Enrichment Analysis analysis associated ATXN3 with immune regulation, interferon signaling, Huntington’s disease pathways, and chromosomal organization (Fig. S6A, Table S4). Notably, JAKs and P53—functioning as an oncogene and tumor suppressor, respectively—were significantly enriched across these relevant pathways (highlighted in red, Table S5). Existing literature shows ATXN3 can stabilize P53 to induce apoptosis [27, 28], while JAK3/STAT5 activation promotes proliferation [35–38]. These opposing mechanisms provide a plausible basis for ATXN3’s subtype-specific functions: P53-mediated suppression in some contexts, and STAT5-driven oncogenesis in others. To investigate this potential mechanism, we analyzed the relationships among HIF-1α, ATXN3, p-STAT5, and P53 in clinical cervical cancer tissues by WB and IHC. In HPV16^+^ SCC, HIF-1α, ATXN3, and p-STAT5 were upregulated, while P53 was downregulated (Fig. 7A, B, F, G). ATXN3 correlated positively with HIF-1α and p-STAT5, but not with P53 (Fig. 7C, H). In HPV18^+^ AC, HIF-1α was elevated, while ATXN3 and P53 were reduced; p-STAT5 remained unchanged (Fig. 7D, E, I-J). Here, ATXN3 correlated negatively with HIF-1α and positively with P53, but not with p-STAT5 (Fig. 7K). These subtype-specific expression and correlation patterns suggest ATXN3 influences progression via p-STAT5 in HPV16^+^ SCC and through P53 in HPV18^+^ AC.

### ATXN3 affects the progression of different subtypes of cervical cancer cells by stabilizing P53 or increasing STAT5 phosphorylation

**Fig. 8:**
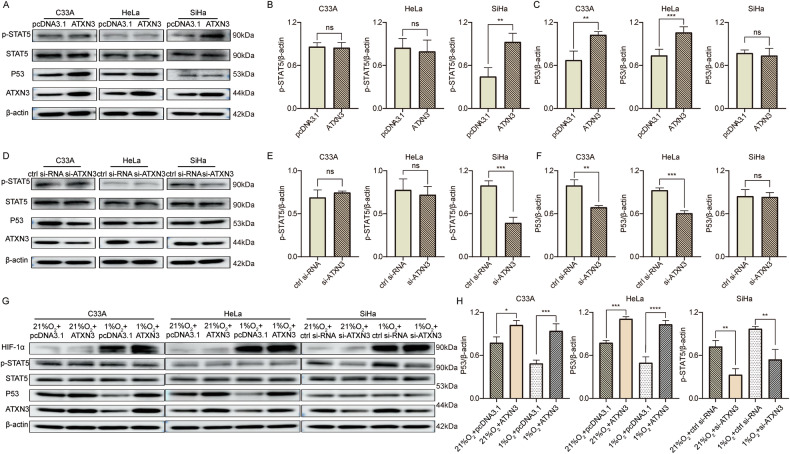
ATXN3 promotes the progression of different subtypes of cervical cancer cells through P53 or p-STAT5. **A** Effect of ATXN3 overexpression on P53 and p-STAT5 protein levels in C33A, HeLa, and SiHa cells. (n ≥ 3 independent cell cultures). **B** Statistical analysis of the effect of ATXN3 overexpression on p-STAT5 protein levels in C33A, HeLa, and SiHa cells. Each individual data point represents an independent cell culture experiment. **C** Statistical analysis of the effect of ATXN3 overexpression on P53 protein levels in C33A, HeLa, and SiHa cells. Each individual data point represents an independent cell culture experiment. **D** Effect of ATXN3 knockdown on P53 and p-STAT5 protein levels in C33A, HeLa, and SiHa cells. (*n* = 3 independent cell cultures). **E** Statistical analysis of the effect of ATXN3 knockdown on p-STAT5 protein levels in C33A, HeLa, and SiHa cells. Each individual data point represents an independent cell culture experiment. **F** Statistical analysis of the effect of ATXN3 knockdown on P53 protein levels in C33A, HeLa, and SiHa cells. Each individual data point represents an independent cell culture experiment. **G** Changes in ATXN3, P53, and p-STAT5 protein levels in C33A, HeLa, and SiHa cells after ATXN3 overexpression or knockdown under hypoxic conditions. (*n* = 3 independent cell cultures). **H** Statistical analysis of changes in ATXN3, P53, and p-STAT5 protein levels in C33A, HeLa, and SiHa cells after ATXN3 overexpression or knockdown under hypoxic conditions. Each individual data point represents an independent cell culture experiment. The statistical notations used in the figure are:ns *P* > 0.05, **P < 0.01, ***P < 0.001, and ****P < 0.0001. Data are represented as mean ± SD. Two-tailed unpaired Student’s t -test was employed for analyses in panels (**B**, **C**, **E** and **F**), while One-way ANOVA were performed for panels (**H**).

To elucidate the subtype-specific mechanisms of ATXN3, we examined its effects in C33A, HeLa, and SiHa cells. While ATXN3 modulation did not alter P53 mRNA levels (Fig. S6C, D), protein analysis revealed distinct regulatory patterns: ATXN3 overexpression increased P53 protein in C33A and HeLa cells without affecting p-STAT5, whereas in SiHa cells it elevated p-STAT5 without changing P53 or total STAT5 (Fig. 8A–C). Knockdown experiments reciprocally reduced P53 in C33A and HeLa, and decreased p-STAT5 specifically in SiHa (Fig. 8D–F). Under hypoxia, P53 was downregulated in C33A and HeLa—an effect reversed by ATXN3 overexpression—while p-STAT5 was upregulated in SiHa and normalized by ATXN3 knockdown (Fig. 8G, H). To understand how ATXN3 enhances STAT5 phosphorylation, molecular docking was performed. As JAK3 is a well-established upstream kinase of STAT5 [39, 40] and has been reported to play a critical role in cervical cancer progression [35–38, 41, 42], we modeled ATXN3’s interaction with phosphorylated JAK3 (p-JAK3, Y980). The results showed strong binding (ΔG = –11.5 kcal/mol) between ATXN3 and p-JAK3, mediated by specific hydrogen bonds and hydrophobic interactions (Supplementary Fig. 7A–B, Table S6). By contrast, ATXN3 binding to STAT5 or p-STAT5 was significantly weaker (Supplementary Fig. 7C, D, Table S6). Collectively, these findings suggest that ATXN3 most preferentially interacts directly with the p-JAK3, rather than with its downstream substrate STAT5, providing a structural biological explanation for the specific upregulation of p-STAT5 levels by ATXN3.

## Discussion

The traditional morphologic classification of cervical cancer (e.g., SCC and AC) has limited prognostic utility. The 2020 WHO Classification incorporates HPV status, enhancing its clinical relevance [43, 44]. Given that HPV16 and 18 cause over 70% of cases—with HPV16 strongly associated with SCC and HPV18 with AC [45–47]—and that HPV-negative tumors, though rare, are more aggressive and increasingly prevalent post-vaccination [43], we investigated three integrated clinicopathological subtypes: HPV16^+^ SCC, HPV18^+^ AC, and HPV⁻ SCC. These were modeled using well-characterized cell lines: SiHa (HPV16^+^ SCC), HeLa (HPV18^+^ AC), and C33A (HPV⁻ SCC), selected for their primary tumor origin, single HPV infection status, and minimal confounding factors [48–50]. Although these models do not encompass full tumor heterogeneity, they offer clinically relevant insights. To elucidate subtype-specific differences—such as the context-dependent regulation of ATXN3 by HIF-1α—we examined interactions among hypoxia, HPV infection, histologic type, and ATXN3. Existing evidence suggests that HPV16 may synergize with HIF-1α to promote malignancy, while E6/E7 proteins impede keratinocyte differentiation [51, 52] Differences in viral tropism and oncogenic potential further underscore the intertwined biology of HPV and histology in cervical cancer, supporting the integrated subtyping approach employed in this study. However, these models cannot fully capture the genetic diversity of actual patient tumors and that expanding the panel of cell lines would improve the generalizability of our findings.

Our study establishes ATXN3 as a context-dependent molecular switch in cervical cancer, showing opposing functions across subtypes: it is upregulated in HPV16^+^ SCC but downregulated in HPV18^+^ AC. Hypoxia further diversifies its regulation—HIF-1α suppresses ATXN3 in C33A and HeLa cells, yet upregulates it in SiHa cells. Functionally, ATXN3 overexpression inhibited malignancy in C33A and HeLa cells but promoted it in SiHa, effects reversible by knockdown. ATXN3 modulation counteracted hypoxia-driven progression in vitro. In vivo experiments also demonstrated that ATXN3 knockdown promoted the growth of C33A/HeLa xenografts while inhibiting that of SiHa. Mechanistically, ATXN3 stabilizes P53 in C33A/HeLa, exerting tumor-suppression, whereas it phosphorylates STAT5, driving oncogenesis in SiHa. This duality aligns with its roles in other cancers—oncogenic in prostate, renal, and breast cancers[22–25, 53–56], but suppressive in colorectal adenocarcinoma[26, 57]. The relatively small sample size of HPV18^+^ AC specimens and absence of other rare subtypes may constrain the generalizability of our findings; and future studies utilizing well-stratified, subtype-specific patient cohorts and long-term follow-up will be of greater clinical significance to clarify the prognostic role of ATXN3. Future work will focus on identifying highly specific ATXN3 modulators and validating their efficacy in subtype-relevant in vivo models.

The regulatory network controlling ATXN3 expression in cervical cancer remains poorly understood. Our study identifies HIF-1α as a key upstream regulator, revealing a striking subtype-specific influence: under hypoxia, HIF-1α knockdown upregulated ATXN3 in C33A (HPV⁻ SCC) and HeLa (HPV18^+^ AC) cells, but downregulated it in SiHa (HPV16^+^ SCC) cells. These results were consistent at both mRNA and protein levels and supported by clinical data, which showed a positive correlation between HIF-1α and ATXN3 in HPV16^+^ SCC specimens and a negative correlation in HPV18^+^ AC tissues. Although HIF-1α is known to activate genes like Mucin 4 and vascular endothelial growth factor by binding HREs [58, 59], it can also suppress others such as P53 and otubain1 through interactions with certain regulatory factors [60, 61]. We propose that HIF-1α may exert dual control over ATXN3 through HRE binding, potentially modulated by subtype-specific co-regulators—a mechanism requiring further investigation.

The downstream signaling network of ATXN3 remains incompletely elucidated, though it is known to exert pleiotropic and context-dependent effects in cancer. Through deubiquitination, ATXN3 modulates key pathways including Hippo (yes-associated protein and transcriptional coactivator with PDZ-binding motif), epigenetic regulators (histone deacetylase3 /histone deacetylase6), and immune-related molecules (programmed death-ligand 1, S100 calcium binding protein A8) [22, 24, 53, 55–57, 62–64]. It can either stabilize oncoproteins like kruppel like factor 4 or tumor suppressors like P53, and even transcriptionally repress tensin homolog [23, 25, 27, 54]. Building on prior reports of ATXN3-mediated P53 stabilization, we study in cervical cancer subtypes. We observed a positive ATXN3-P53 protein correlation in HPV18^+^ AC, but not in HPV16^+^ SCC—likely due to HIF-1α-mediated suppression of P53 or HPV16 E6/E6AP-mediated P53 degradation[60, 65, 66]. Functional assays showed that ATXN3 regulates P53 post-translationally in C33A and HeLa cells—protein levels varied with ATXN3 expression without changes in mRNA, counteracting hypoxia-induced degradation. In SiHa (HPV16^+^ SCC) cells, however, P53 remained unaffected by ATXN3 or hypoxia, indicating subtype-specific mechanisms. Future studies should validate the specific deubiquitination site through which ATXN3 stabilizes P53 in cervical cancer. Given that HPV E6/E7 oncoproteins promote P53 degradation, they may also influence the ATXN3-P53 axis [30, 67, 68]. We hypothesize that ATXN3-mediated deubiquitination could counteract HPV-induced P53 degradation, particularly in HPV18^+^ AC, potentially suppressing tumor progression. These mechanisms, however, remain speculative and require experimental confirmation.

Our study reveals a subtype-specific role for ATXN3 in regulating STAT5 signaling in cervical cancer. In HPV16^+^ SCC, p-STAT5—but not total STAT5—was elevated and positively correlated with ATXN3 expression. This pattern was absent in HPV18^+^ AC. In SiHa cells (HPV16^+^ SCC), ATXN3 specifically modulated p-STAT5 levels without affecting total STAT5, and ATXN3 knockdown reversed hypoxia-induced STAT5 phosphorylation. No such regulation occurred in C33A or HeLa cells. Molecular docking suggested strong binding between ATXN3 and phosphorylated JAK3, indicating that ATXN3 may stabilize active JAK3 to enhance STAT5 phosphorylation. Given that HPV oncoproteins like E7 are known to promote STAT5 activation [69–72], we hypothesize that in HPV16^+^ SCC, ATXN3 may facilitate tumor progression through the JAK3/STAT5 pathway. This mechanism, however, requires further experimental validation.

In conclusion, our study establishes ATXN3 as a clinically relevant biomarker and context-dependent regulator in cervical cancer pathogenesis. We have elucidated a dual mechanistic paradigm wherein: in HPV18^+^ AC and HPV⁻ SCC, hypoxia-mediated HIF-1α activation suppresses ATXN3 expression, leading to decreased P53 stability and consequent tumor progression; while in HPV16^+^ SCC, hypoxia-induced HIF-1α upregulates ATXN3 expression, which drives oncogenesis through STAT5 phosphorylation—likely mediated through stabilization of its upstream kinase p-JAK3. These subtype-specific pathways provide a molecular rationale for ATXN3’s opposing functional roles across cervical cancer subtypes. This work thus provides a foundational framework for developing precision therapeutic strategies tailored to individual cervical cancer subtypes based on their distinct ATXN3 regulatory networks.

## Materials and methods

### Ethics and clinical sample collection

Cervical cancer tissues and paired adjacent non-tumor samples were collected from Qinghai University Affiliated Hospital (June 2024–February 2025), including 21 HPV16^+^ SCC and 4 HPV18^+^ AC cases. Patients with prior radiotherapy, chemotherapy, or immunotherapy were excluded. After informed consent was obtained, clinical data were collected. Sample size was determined by preliminary effect size and subtype prevalence, with HPV16^+^ SCC prioritized for statistical power and HPV18^+^ AC considered exploratory. The study was approved by the Ethics Committee of Qinghai University Medical College (PJ202401-11).

### Immunohistochemistry (IHC)

IHC was performed using a poly-HRP Anti-mouse/rabbit IgG detection kit (E-IR-R217, Proteintech, Wuhan, China). Formalin-fixed, paraffin-embedded tissues were sectioned at 4 μm, dewaxed, rehydrated, and subjected to antigen retrieval. After blocking endogenous peroxidase and non-specific binding, sections were incubated overnight at 4 °C with primary antibodies against HIF-1α (1:200, 36169S, Cell Signaling Technology, Danvers, Massachusetts, USA), ATXN3 (1:200, MAB5360, Sigma, Hesse, Germany), p-STAT5 (1:100, 80115-1-RR, Proteintech), and P53 (1:100, 10442-1-AP, Proteintech). HRP-conjugated secondary antibodies were applied for 50 min at room temperature, followed by diaminobenzidine development and hematoxylin counterstaining. All slides were washed with phosphate-buffered saline (PBS) between steps and evaluated by experienced pathologists for staining intensity and positive cell percentage.

### Cell culture

The human cervical cancer cell lines C33A (IMMO-251545), HeLa (IMMO-241373), and SiHa (IMMO-251426) were purchased from Xiamen Immocell Biotechnology. All cell lines were authenticated by short tandem repeat profiling and confirmed to be free of mycoplasma contamination. All cells were cultured in Dulbecco’s Modified Eagle Medium (#C11995500BT, Gibco, New york, USA) with 10% fetal bovine serum (#10099141 C, Gibco), penicillin (100 U/mL), and streptomycin (100 μg/mL) (#10378016, Gibco). The incubator (150i, Thermo Fisher Scientific, Waltham, USA) was maintained with 5% CO_2_ at 37 °C. Oxygen concentrations in normoxic and hypoxia cultures were 21% and 1%, respectively.

### Cell transfection

The DNA sequence encoding ATXN3 was cloned into the pcDNA3.1 vector for transient expression. The siRNA duplexes were chemically synthesized by Sangon Biotech. Transfection was performed according to the instructions for Lipofectamine 2000 Transfection Reagent (Invitrogen, USA, Waltham, USA) and Lipofectamine RNAiMAX Reagent (Invitrogen, USA). siRNA sequences used in this study were as follows:

*Control siRNA sense:* 5ʹ- UUCUCCGAACGUGUCACGUdTdT-3ʹ

*ATXN3 siRNA sense:* 5ʹ- GGACAGAGUUCACAUCCAUdTdT-3ʹ

*HIF-1α* siRNA sense: 5ʹ- CUGAUGACCAGCAACUUGAdTdT -3ʹ

### Quantitative real-time polymerase chain reaction

Total RNA was extracted from cervical cancer cells and tissues using Trizol reagent (Invitrogen, USA). All primer sequences were synthesized by Sangon Biotech. The two-step cDNA reverse transcription was performed using the HiScript II Q RT SuperMix for qPCR (+gDNA wiper) (#R223-01, Vazyme, Nanjing, China) reagent. Finally, 2× RealStar Green Power Mixture (#A311-10 Genstar, Winnipeg, Canada) was utilized to perform quantitative real-time polymerase chain reaction. Expression data were normalized to the endogenous reference gene ribosomal protein L13a to control for variability in expression levels. Relative quantification calculations were performed using the 2^−ΔΔCT^ method. Primer sequences used in this study were as follows:

*RPL13A-F*: 5ʹ- GCCATCGTGGCTAAACAGGTA-3’

*RPL13A-R*: 5ʹ- GTTGGTGTTCATCCGCTTGC-3’

*ATXN3-F*: 5ʹ-AGCAGCAAAAGCAGCAAC-3’

*ATXN3-R*: 5ʹ-TAGCGAACATGATGAATG-3’

*HIF-1α-F: 5* *ʹ-GCCGCTGGAGACACAATCAT-3ʹ*

*HIF-1α-R: 5’-GAAGTGGCTTTGGCGTTTCA-3’*

*P53-F*: 5ʹ-GAACAGCTTTGAGGTGCGTG-3ʹ

*P53-R*: 5ʹ-CTTCTTTGGCTGGGGAGAGG-3ʹ

### Western blot(WB)

Total proteins were extracted from cells or tissues using the radioimmunoprecipitation assay lysis buffer (Invitrogen, USA). Primary antibodies targeting HIF-1α (1:1000, 36169S, Cell Signaling Technology), ATXN3 (1:1000, MAB5360, Sigma), p-STAT5 (1:1000, #9314 T, Cell Signaling Technology), STAT5 (1:1000, #25656 T, Cell Signaling Technology), P53 (1:1000, #2527 T, Cell Signaling Technology), and β-actin (1:1000, 66009-1-Ig, Proteintech) were used to specifically bind to the corresponding antigens. Then, the samples were incubated with horseradish peroxidase-conjugated secondary antibodies derived from rabbit (1:8000, SA00001-2, Proteintech) or mouse (1:8000, SA00001-1, Proteintech). The protein bands were detected using a chemiluminescence instrument (Amersham Imager 600, GE, Boston, USA) after applying the extracellular loop Western-Blotting Substrate reagent (Original western blots).

### Cell proliferation by MTS

Cells were seeded at 1 × 10^4^ per well in 96-well plates (Corning, 3599, USA). After attachment, MTS assays were conducted daily for 5 days using the CellTiter 96® AQueous One Solution Reagent (#G3580, Promega, Madison, USA). For each measurement, 20 μL of reagent was added to 100 μL medium per well, followed by incubation at 37 °C with 5% CO₂ for 2 h. Absorbance at 490 nm was measured using a microplate reader (Tecan, Männedorf, Switzerland) after brief shaking.

### Colony formation

Cells were seeded uniformly at 2000 per well in 6-well plates (Corning, 3516) and cultured for 1–2 weeks. Colonies were fixed with 4% paraformaldehyde, stained with 1% crystal violet, and washed with PBS between steps. Clusters containing more than 50 cells were counted using ImageJ.

### Migration and invasion

Cell migration and invasion assays were conducted using 24-well Transwell chambers (Corning, 3422). For invasion assays, chambers were pre-coated with Matrigel (Corning, #356234). In total, 1 × 10^6^ cells in 200 μL serum-free medium were seeded in the upper chamber, and 600 μL complete medium was added to the lower well. After 24 h, cells were fixed with 4% paraformaldehyde, stained with crystal violet, and imaged under a microscope (ZEISS). The number of migrated or invaded cells was quantified from five random fields using ImageJ.

### Wound healing assay

A wound-healing assay was performed by seeding 1 × 10^6^ cells per well in 6-well plates (Corning, 3516). At full confluence, a sterile pipette tip was used to create scratches. After rinsing with PBS to remove debris, cells were cultured in medium with 2% fetal bovine serum. Wound closure was monitored using microscopy (ZEISS) and quantified with Photoshop.

### Database mining

Gene sequences were obtained from the National Center for Biotechnology Information (Gene ID: 4287). Putative transcription factor binding sites were predicted using JASPAR (https://jaspar.elixir.no/ [73]). Functional annotation of ATXN3 was performed using Gene Ontology and Kyoto Encyclopedia of Genes and Genomes analyses via the “clusterProfiler” R package (*p* < 0.05). Gene Set Enrichment Analysis identified pathways correlated with ATXN3 expression (|NES | > 1, nominal *p* < 0.05, false discovery rate (FDR) < 0.25). Protein structures for ATXN3 (P54252), STAT5 (P42229), phosphorylated STAT5 (Y694) (P42229), and phosphorylated JAK3 (Y980) (P52333) were retrieved from the PDB (https://www.rcsb.org/). Protein-protein docking was performed using GRAMM-X (http://vakser.bioinformatics.ku.edu/resources/gramm/grammx) [74], and complexes were visualized using PyMOL(Version 2.5.2).

### Orthotopic xenograft tumor model

The animal study was approved by the Ethics Committee of Qinghai University Medical College (PJ202401-11). Female sterile BALB/c nude mice (4–6 weeks old) were acclimatized for one week under specific pathogen-free conditions. Animals were randomly assigned to control or experimental groups using a spreadsheet-based procedure. C33A, HeLa, and SiHa cells transfected with control or ATXN3-targeting siRNA were resuspended in Matrigel/PBS (1:1, 3 × 10^7^ cells/ml), and 100 µL was injected subcutaneously per mouse. Tumor volume was measured every two days using V = (a × b²)/2. Mice were euthanized when tumors reached ~1500 mm³; tumors were excised and weighed. Group sizes (n = 6 per group) followed standard guidelines (NC3Rs) and common preclinical practice. Animals were excluded for predefined reasons including failed engraftment, severe non-tumor morbidity, unmet humane endpoints, technical errors, or non-treatment mortality. Investigators were blinded to group assignments throughout the experiment and data collection.

### Statistical analysis

All statistical analyses were performed using GraphPad Prism 8.0. Normality and homogeneity of variances were assessed. Data are presented as “mean ± standard deviation (SD)”. Comparisons between two groups used Student’s *t* test or Mann–Whitney U test (for small samples). Multiple groups were compared with one-way or two-way ANOVA. Correlations were evaluated by linear regression.“P-value < 0.05” was considered statistically significant.

## Supplementary information

Supplementary Table 1

Supplementary Table 2

Supplementary Table 3

Supplementary Table 4

Supplementary Table 5

Supplementary Table 6

Supplementary Figure 1

Supplementary Figure 2

Supplementary Figure 3

Supplementary Figure 4

Supplementary Figure 5

Supplementary Figure 6

Supplementary Figure 7

Supplementary table legend

Supplementary figure legend

Supplementary File

Original western blots

## Data Availability

The data that support the findings of this study are available from the corresponding author, upon reasonable request.
