# Supplementary material for: Hypoxia promotes progression of cervical cancer by modulating the ATXN3-enhanced P53 stability or STAT5 phosphorylation
Source: Cell Death Discov. 2026 Jan 8;12:4. doi: 10.1038/s41420-025-02822-0 (PMC12783129; doi:10.1038/s41420-025-02822-0)
Supplement: Supplementary file 1 — Supplementary Table 1 [file 41420_2025_2822_MOESM1_ESM.docx]

Supplementary Table 1: Recognition sequences of HIF-1α obtained from the JASPAR database.Species: [Homo sapiens](https://www.ncbi.nlm.nih.gov/Taxonomy/Browser/wwwtax.cgi?mode=Info&id=9606)

(JASPAR http://jaspar.genereg.net/)

|  | **MA0259.1** | **MA0259.2** | **MA1106.1** | **MA1106.2** |
| --- | --- | --- | --- | --- |
| Sequence logo | 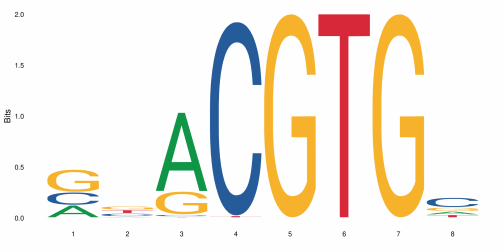 | 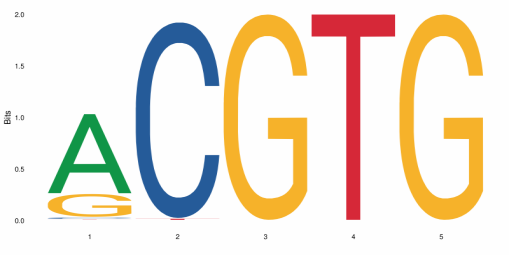 | 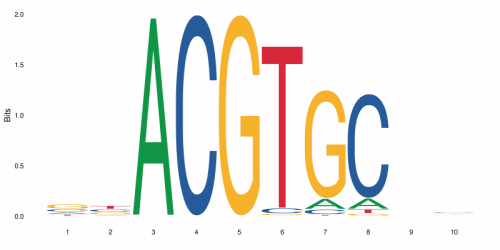 | 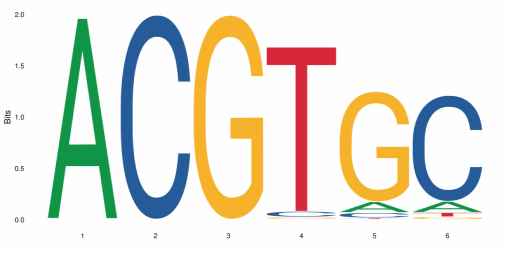 |
| Frequency matrix | A [ 27 10 78 0 0 0 0 18 ] | A [ 78 0 0 0 0 ] | A [ 197 105 976 0 1 2 84 88 206 164 ] | A [ 976 0 1 2 84 88 ] |
|  | C [ 28 29 2 103 0 0 0 51 ] | C [ 2 103 0 0 0 ] | C [ 229 270 0 979 0 33 41 837 290 315 ] | C [ 0 979 0 33 41 837 ] |
|  | G [ 49 34 23 0 104 0 104 20 ] | G [ 23 0 104 0 104 ] | G [ 414 263 2 0 979 11 844 20 257 271 ] | G [ 2 0 979 11 844 20 ] |
|  | T [ 0 31 1 1 0 104 0 15 ] | T [ 1 1 0 104 0 ] | T [ 140 342 2 1 0 934 11 35 227 230 ] | T [ 2 1 0 934 11 35 ] |
