# Supplementary material for: Hypoxia promotes progression of cervical cancer by modulating the ATXN3-enhanced P53 stability or STAT5 phosphorylation
Source: Cell Death Discov. 2026 Jan 8;12:4. doi: 10.1038/s41420-025-02822-0 (PMC12783129; doi:10.1038/s41420-025-02822-0)
Supplement: Supplementary file 2 — Supplementary Table 2 [file 41420_2025_2822_MOESM2_ESM.docx]

Supplementary Table 2: ATXN3 promoter—HIF-1α target sequences. Threshold:0.85

(JASPAR http://jaspar.genereg.net/)

| **Matrix ID** | **Name** | **Score** | **Relative score** | **Sequence ID** | **Start** | **End** | **Strand** | **Predicted sequence** |
| --- | --- | --- | --- | --- | --- | --- | --- | --- |
| [MA0259.1](https://jaspar.elixir.no/matrix/MA0259.1) | MA0259.1.ARNT::HIF1A | 10.231993 | 0.9711507 | NC_000014.9:c92108582-92106482 | 1911 | 1918 | - | CCACGTGC |
| [MA0259.1](https://jaspar.elixir.no/matrix/MA0259.1) | MA0259.1.ARNT::HIF1A | 9.739024 | 0.9564442 | NC_000014.9:c92108582-92106482 | 1911 | 1918 | + | GCACGTGG |
| [MA0259.1](https://jaspar.elixir.no/matrix/MA0259.1) | MA0259.1.ARNT::HIF1A | 8.802208 | 0.9284967 | NC_000014.9:c92108582-92106482 | 1837 | 1844 | - | ACACGTGA |
| [MA0259.1](https://jaspar.elixir.no/matrix/MA0259.1) | MA0259.1.ARNT::HIF1A | 8.739632 | 0.9266299 | NC_000014.9:c92108582-92106482 | 722 | 729 | - | AGGCGTGC |
| [MA0259.1](https://jaspar.elixir.no/matrix/MA0259.1) | MA0259.1.ARNT::HIF1A | 8.664086 | 0.92437625 | NC_000014.9:c92108582-92106482 | 1902 | 1909 | + | CTGCGTGC |
| [MA0259.1](https://jaspar.elixir.no/matrix/MA0259.1) | MA0259.1.ARNT::HIF1A | 8.294679 | 0.9133559 | NC_000014.9:c92108582-92106482 | 1391 | 1398 | - | GGGCGTGG |
| [MA0259.1](https://jaspar.elixir.no/matrix/MA0259.1) | MA0259.1.ARNT::HIF1A | 8.171119 | 0.90966976 | NC_000014.9:c92108582-92106482 | 149 | 156 | - | GTGCGTGG |
| [MA0259.1](https://jaspar.elixir.no/matrix/MA0259.1) | MA0259.1.ARNT::HIF1A | 7.6062136 | 0.8928173 | NC_000014.9:c92108582-92106482 | 277 | 284 | - | AAACGTGG |
| [MA0259.1](https://jaspar.elixir.no/matrix/MA0259.1) | MA0259.1.ARNT::HIF1A | 7.5398693 | 0.8908381 | NC_000014.9:c92108582-92106482 | 1997 | 2004 | + | CGGCGTGG |
| [MA0259.1](https://jaspar.elixir.no/matrix/MA0259.1) | MA0259.1.ARNT::HIF1A | 7.368294 | 0.88571954 | NC_000014.9:c92108582-92106482 | 65 | 72 | + | ATGCGTGG |
| [MA0259.1](https://jaspar.elixir.no/matrix/MA0259.1) | MA0259.1.ARNT::HIF1A | 7.357862 | 0.88540834 | NC_000014.9:c92108582-92106482 | 588 | 595 | - | AGGCGTGA |
| [MA0259.1](https://jaspar.elixir.no/matrix/MA0259.1) | MA0259.1.ARNT::HIF1A | 6.1745734 | 0.85010797 | NC_000014.9:c92108582-92106482 | 1091 | 1098 | + | CTCCGTGC |
| [MA0259.2](https://jaspar.elixir.no/matrix/MA0259.2) | MA0259.2.ARNT::HIF1A | 9.08275 | 1 | NC_000014.9:c92108582-92106482 | 278 | 282 | - | ACGTG |
| [MA0259.2](https://jaspar.elixir.no/matrix/MA0259.2) | MA0259.2.ARNT::HIF1A | 9.08275 | 1 | NC_000014.9:c92108582-92106482 | 1838 | 1842 | - | ACGTG |
| [MA0259.2](https://jaspar.elixir.no/matrix/MA0259.2) | MA0259.2.ARNT::HIF1A | 9.08275 | 1 | NC_000014.9:c92108582-92106482 | 1839 | 1843 | + | ACGTG |
| [MA0259.2](https://jaspar.elixir.no/matrix/MA0259.2) | MA0259.2.ARNT::HIF1A | 9.08275 | 1 | NC_000014.9:c92108582-92106482 | 1912 | 1916 | - | ACGTG |
| [MA0259.2](https://jaspar.elixir.no/matrix/MA0259.2) | MA0259.2.ARNT::HIF1A | 9.08275 | 1 | NC_000014.9:c92108582-92106482 | 1913 | 1917 | + | ACGTG |
| [MA0259.2](https://jaspar.elixir.no/matrix/MA0259.2) | MA0259.2.ARNT::HIF1A | 7.42617 | 0.9363618 | NC_000014.9:c92108582-92106482 | 67 | 71 | + | GCGTG |
| [MA0259.2](https://jaspar.elixir.no/matrix/MA0259.2) | MA0259.2.ARNT::HIF1A | 7.42617 | 0.9363618 | NC_000014.9:c92108582-92106482 | 150 | 154 | - | GCGTG |
| [MA0259.2](https://jaspar.elixir.no/matrix/MA0259.2) | MA0259.2.ARNT::HIF1A | 7.42617 | 0.9363618 | NC_000014.9:c92108582-92106482 | 589 | 593 | - | GCGTG |
| [MA0259.2](https://jaspar.elixir.no/matrix/MA0259.2) | MA0259.2.ARNT::HIF1A | 7.42617 | 0.9363618 | NC_000014.9:c92108582-92106482 | 723 | 727 | - | GCGTG |
| [MA0259.2](https://jaspar.elixir.no/matrix/MA0259.2) | MA0259.2.ARNT::HIF1A | 7.42617 | 0.9363618 | NC_000014.9:c92108582-92106482 | 1287 | 1291 | + | GCGTG |
| [MA0259.2](https://jaspar.elixir.no/matrix/MA0259.2) | MA0259.2.ARNT::HIF1A | 7.42617 | 0.9363618 | NC_000014.9:c92108582-92106482 | 1392 | 1396 | - | GCGTG |
| [MA0259.2](https://jaspar.elixir.no/matrix/MA0259.2) | MA0259.2.ARNT::HIF1A | 7.42617 | 0.9363618 | NC_000014.9:c92108582-92106482 | 1515 | 1519 | + | GCGTG |
| [MA0259.2](https://jaspar.elixir.no/matrix/MA0259.2) | MA0259.2.ARNT::HIF1A | 7.42617 | 0.9363618 | NC_000014.9:c92108582-92106482 | 1904 | 1908 | + | GCGTG |
| [MA0259.2](https://jaspar.elixir.no/matrix/MA0259.2) | MA0259.2.ARNT::HIF1A | 7.42617 | 0.9363618 | NC_000014.9:c92108582-92106482 | 1999 | 2003 | + | GCGTG |
| [MA1106.1](https://jaspar.elixir.no/matrix/MA1106.1) | MA1106.1.HIF1A | 11.699058 | 0.96758515 | NC_000014.9:c92108582-92106482 | 1909 | 1918 | - | CCACGTGCCG |
| [MA1106.1](https://jaspar.elixir.no/matrix/MA1106.1) | MA1106.1.HIF1A | 8.560859 | 0.89369357 | NC_000014.9:c92108582-92106482 | 1835 | 1844 | - | ACACGTGACC |
| [MA1106.1](https://jaspar.elixir.no/matrix/MA1106.1) | MA1106.1.HIF1A | 7.6497073 | 0.87223965 | NC_000014.9:c92108582-92106482 | 1911 | 1920 | + | GCACGTGGGC |
| [MA1106.1](https://jaspar.elixir.no/matrix/MA1106.1) | MA1106.1.HIF1A | 6.9284344 | 0.8552567 | NC_000014.9:c92108582-92106482 | 1837 | 1846 | + | TCACGTGTCC |
| [MA1106.1](https://jaspar.elixir.no/matrix/MA1106.1) | MA1106.1.HIF1A | 6.9258766 | 0.8551964 | NC_000014.9:c92108582-92106482 | 1821 | 1830 | + | GGACGCGCTA |
| [MA1106.2](https://jaspar.elixir.no/matrix/MA1106.2) | MA1106.2.HIF1A | 11.279793 | 1 | NC_000014.9:c92108582-92106482 | 1911 | 1916 | - | ACGTGC |
| [MA1106.2](https://jaspar.elixir.no/matrix/MA1106.2) | MA1106.2.HIF1A | 8.139631 | 0.9172273 | NC_000014.9:c92108582-92106482 | 1837 | 1842 | - | ACGTGA |
| [MA1106.2](https://jaspar.elixir.no/matrix/MA1106.2) | MA1106.2.HIF1A | 7.1549625 | 0.891272 | NC_000014.9:c92108582-92106482 | 375 | 380 | + | ACGTCC |
| [MA1106.2](https://jaspar.elixir.no/matrix/MA1106.2) | MA1106.2.HIF1A | 6.9777055 | 0.8865997 | NC_000014.9:c92108582-92106482 | 1839 | 1844 | + | ACGTGT |
| [MA1106.2](https://jaspar.elixir.no/matrix/MA1106.2) | MA1106.2.HIF1A | 6.7519007 | 0.88064754 | NC_000014.9:c92108582-92106482 | 1823 | 1828 | + | ACGCGC |
| [MA1106.2](https://jaspar.elixir.no/matrix/MA1106.2) | MA1106.2.HIF1A | 6.3556566 | 0.87020284 | NC_000014.9:c92108582-92106482 | 277 | 282 | - | ACGTGG |
| [MA1106.2](https://jaspar.elixir.no/matrix/MA1106.2) | MA1106.2.HIF1A | 6.3556566 | 0.87020284 | NC_000014.9:c92108582-92106482 | 1913 | 1918 | + | ACGTGG |
