# Supplementary material for: Hypoxia promotes progression of cervical cancer by modulating the ATXN3-enhanced P53 stability or STAT5 phosphorylation
Source: Cell Death Discov. 2026 Jan 8;12:4. doi: 10.1038/s41420-025-02822-0 (PMC12783129; doi:10.1038/s41420-025-02822-0)
Supplement: Supplementary file 3 — Supplementary Table 3 [file 41420_2025_2822_MOESM3_ESM.docx]

Supplementary Table 3: Human sequence of ATXN3 promoter

(https://www.ncbi.nlm.nih.gov/)

| ATXN3 promoter sequence chr14: 92106482-chr14: 92108582 |
| --- |
| >NC_000014.9:c92108582-92106482 Homo sapiens chromosome 14, GRCh38.p14 Primary Assembly  TGCATTTGCATATTAAAAGGCTAGGGTGGAATGGCCAGTTTTATTAGGGGGCTACATGAATGACATGCGTGGTCAAACCAGTCCCCTGAGCCCTATGCAAATCAGACACCGCCTCCTGCATCCTCCTCCTATAACTGGCTGGTATCTCCCACGCACTCTGGGTCTCCTCTCTCAGCTTTGGAGCACCCATCCCTCTGTCTCTGTACAGGGGAGCTGCTTCTTTCTTTCTTCTCCCTTCCTTCTTGCTTATTAAACTCTCCGCTCCTTAAAACCACTCCACGTTTGTCCATGTCGTTTTATGTAATTCGACTCAAGACGAAAAAGGCTAGTATTCCTCCACTCGGTATCATCACCACATTCTGTTGAGTCTCTTTACGTCCAAAGAATCTCTCAAAATGGGCTATTTCCATCTTTAAGACACTCTCTTAAACCACCATCGTCTCTCCCAAGATTCCTTCAACAGCAACTGCTTTTTTCTGCAGCCAGTGATCTTTCTAAAAGGCAAACATGACCACATGTCTTCTTTTAAAATATTTATATAGCTCCCCACATCCTTTAAAAGGTACACTTTGGCCAGGTGCGGTGGCTCACGCCTGTAATTCCAGGACTTTGGGAGGCCAAGGCAGGTGGATCACCTGAGGCTGGGGGTTCGAGACCAGCCTGGCCAACATGGTACAACCTCTTCTCTACTAAGAATACAAAAATAGTTGGACTTCGTGGTGCACGCCTGTAATCCCAGCTGTTTGGGAGGCTGAGGCAAGAGAATCACTTGAACCCAGGAAGCAGAAGTTGCAGTGAGCGGGGAGATTGTGCCACTGCACTCCAGCCTGGGGGACAGAGAGAGGGCCGTCTCAAAAAAAAAAAAAAAAAAAGTACACCTTAATCTGGTTTCAAGGTGCAAGATCTGCACCTATCCTTCCATTTAAGTTTCATCAAACCGCTGCTACCTCCCCGTCTCCCACACAATTTATGGGACTTCTAAGTTCCCTCTAAAGGGTCCGAACACCTACACTGGTAACAAGCCACCTGGATTTGAATCCTGGCAAGACAACTTACTATCTGACCTTGGACATTGTGCTGTTCTTAACCTCTCCGTGCCTCGGTTTCCTCATGTGTATGAATAACATCAACACCTACATCAAAGTTTGCTGTATTAAATTTGATAATATATGCAAAGCATTTAGAAAAGTGCCTAGCTCATAGAAAGCCTTATGTAAATATTAACTATCATTTTTTTTCTTTTTTGGGGTGGTGGGGGAGGGGTTTCGCTTTTGTTGCCCAGGCTGGCGTGCAATGGCACGATCTCGGCTCACCACAACCTCGGCCTCCCGGGTTCAAGCGATTCTCCTGCCTCAGCCTCCCGAGTACCTGGGATTACAGGCATGCACCACCACGCCCGGGTAATTTTGTATTTTTAGTGGAGACGGGGTTTCTCCATGTTGGTCAGGCTGATCTCAAACTCCCGACCTCAGGTGATCCGCCCGCCTCGGCCTCCCAAAGTGCTGGGATTACAAGCGTGAAGCACCTCGCCCGGCCTAGCTATCATTTTTATACAAGTGCTGGGTTTTGGGAGAATGTAATGATGGCTTTTTTCTTACTAAACTTTCAGTGCAGGAGGAGGAGAAAGAAAGTAAATAGTTATATGAACACAGTAGAAAGTCAAAGTGGAAAACAAAAAGAACATAGAACCCAGGTGAGCGGTCCAGACCTCCCCCCAGAAACCTAAGAATCCATAGAAATGGGTGGGAAGCGGAGAAGATCCTCCAGACAGCAGGTGGCGATGTAGCATCCCCCAGAAGGCCCGCTAACAGAAGCTAGGAGGACGCGCTACCAAGGTCACGTGTCCCCGGCGTTCACTCGCTCTTCGCTTCACGACACTCGCATCCTCACGGGTGATTGGTCTGCGTGCGGCACGTGGGCGGGGTACCGGGGCGGGCCGGGGAGGGGCGGGGTGGGCGGAGGAGAGGGGCAGGGGGCGGAGCTGGAGGGGGTGGTTCGGCGTGGGGGCCGTTGGCTCCAGACAAATAAACATGGAGTCCATCTTCCACGAGAAAGTGAGTGTCCGCGTTCGGTGGGGAGCTGTCTGCCGCGCGGTGGCGGG |
