# Supplementary material for: Hypoxia promotes progression of cervical cancer by modulating the ATXN3-enhanced P53 stability or STAT5 phosphorylation
Source: Cell Death Discov. 2026 Jan 8;12:4. doi: 10.1038/s41420-025-02822-0 (PMC12783129; doi:10.1038/s41420-025-02822-0)
Supplement: Supplementary file 5 — Supplementary Table 5 [file 41420_2025_2822_MOESM5_ESM.docx]

Supplementary Table 5: cancer-related genes were systematically annotated and classified within significantly enriched pathways.(https://www.gsea - msigdb.org/gsea/msigdb/index.jsp;source species:Homo sapiens;contributor :Kyoto Encyclopedia of Genes and Genomes;all collection)

|  |  |  | cytokines and growth factors | oncogenes | tumor suppressors |
| --- | --- | --- | --- | --- | --- |
| high ATXN3 | KEGG | ALLOGRAFT REJECTION | IL2/CD40LG/FASLG/IL4/IL10/1L5/IFNG/IL12B/IL12A/TNF | IL2 | FAS/PRF1 |
|  |  | ADHESION MOLECULES CAMS | CD40LG |  | CDH1 |
|  |  | CYTOKINE CYTOKINE RECEPTOR INTERACTION | CNTF CSF3 CSF2 CSF1 EPO CCL4L2CXCL13 TNF CXCL14 CXCL16 TNFSF10 IL12B TNFSF11 IL12A PDGFRB PDGFRA I IFNA13 I IFNA14 IFNA16 HGF IFNA17 FLT3LG IL23A IL6ST IFNA10 CCL3L3 PDGFB PDGFA TNFRSF11B PDGFC IFNA21 TNFSF18 TGFB2 TGFB1 TSLP TNFSF14 TGFB3 TNFSF15 TNFSF12 TNFSF13  LIF INHBB INHBA PPBP INHBC BMP7 GDF5 IFNW1 IL2 INHBE GH2 IL4 GH1 IL3 CXCL10 IL6 CXCL11 BMP2 IL5 CXCL12  IL7 TNFSF4 IL9 TNFSF9 TNFSF8 IFNA5 IL21 IFNA4 IL22 CXCL6 IFNA7 CXCL9 IFNA6 CXCL8 IL20 IFNA1 IL25 IL26 IL24 IFNA2 CXCL1 FASLG PRL CXCL3 CXCL2 IFNA8 CXCL5 CX3CL1 TNFSF13B AMH PF4V1 IL10 IL11 IL15 PPBPP1 IL13 IL18 IL19 IL1A IFNG IFNE IL1B LTA IFNK XCL2 XCL1 LTB CCL14 CCL13 CTF1  CCL11 IFNL2 IFNL1 CCL8 CCL7 CCL5 CCL4 CCL3 CCL2 CCL1 CCL19 CCL18 IFNL3 CCL17 CCL16 CCL15 CCL25 CCL24 CCL23 CCL22 CCL21 EGF IFNB1 CCL20 CD70 OSM VEGFB VEGFC VEGFD VEGFA KITLG CD40LG CLCF1 LEP CCL28 CCL27 IL17B CCL26 IL17A PF4 | PDGFRB PDGFRA FLT3 MPL PDGFB LIFR EGFR IL2 KIT KDR IL21R TNFRSF17 IL6ST MET CRLF2 | FAS/BMPR1A |
|  |  | GRAFT VERSUS HOST DISEASE | IL1A IL6 IFNG IL1B FASLG TNF IL2 | IL2 | FAS/PRF1 |
|  |  | PRIMARY IMMUNODEFICIENCY | CD40LG | CD79A CIITA LCK JAK3 | 0 |
|  | GO | GRANULOCYTE CHEMOTAXIS | CXCL6 CXCL9 CXCL8 CSF1 MSTN CCL4L2 CXCL17 CXCL1 CXCL13  CXCL3 CXCL2 CXCL5 CX3CL1 MDK PF4V1 EDN1 EDN2 EDN3 IL23A IL1B XCL2 XCL1 SCG2 CCL14 CCL13 CCL11 CCL3L3 CCL8 CCL7 CCL5 CCL4 CCL3 CCL2 CCL1 CCL19 SLIT2 CCL18 CCL16 CCL15  CCL25 CCL24 CCL23 TGFB2 CCL22 CCL21 IL34 PPBP CXCL10 CXCL11 SAA1 CKLF CCL26 PF4 | CD74/SYK | 0 |
|  |  | GRANULOCYTE MIGRATION | CXCL6 CXCL9 CXCL8 CSF1 MSTN CCL4L2 CXCL17 CXCL1 CXCL13 CXCL3 CXCL2CXCL5 CX3CL1 MDK PF4V1 EDN1 EDN2 EDN3 IL1A IL23A IL1B XCL2XCL1 SCG2 CCL14 CCL13 CCL11 CCL3L3 CCL8 CCL7 CCL5 CCL4 CCL3 CCL2 CCL1 CCL19 SLIT2 CCL18 CCL16 CCL15 CCL25 CCL24 CCL23 TGFB2 CCL22 CCL21 IL34 PPBP  CXCL10 CXCL11 SAA1 CKLF CCL26 IL17A PF4 | CD74/SYK | 0 |
|  |  | NEUTROPHIL CHEMOTAXIS | CXCL6 CXCL9 CXCL8CXCL1 CXCL13 CXCL3 CXCL2 CXCL5 CX3CL1 MDK CCL3 CCL19 SLIT2 PF4V1 EDN1 TGFB2 EDN2 EDN3 CCL21  PPBP CXCL10 CXCL11 IL23A IL1B SAA1 XCL1 CKLF PF4 | CD74/SYK | 0 |
|  |  | NEUTROPHIL MIGRATION | CXCL6 CXCL9 CXCL8 CXCL1 CXCL13 CXCL3 CXCL2 CXCL5 CX3CL1 MDK CCL3 CCL19 SLIT2 PF4V1 EDN1 TGFB2 EDN2 EDN3 CCL21  PPBP IL1A CXCL10 CXCL11 IL23A IL1B SAA1 XCL1 CKLF PF4 | CD74/SYK | 0 |
|  |  | RESPONSE TO TYPE II INTERFERON | EDN1 IFNG CCL5 CCL3 IL12B CCL2 FASLG CRIPTO TNF CXCL16 | CD74 CIITA PIM1  PPARG JAK2 RAF1 JAK1 | TP53 |
| low ATXN3 | KEGG | KEGG_BASE_EXCISION_REPAIR | 0 | 0 | MUTYH |
|  |  | KEGG_CITRATE_CYCLE_TCA_CYCLE | 0 | IDH1/IDH2 | FH SDHC SDHD SDHB |
|  |  | KEGG_DNA_REPLICATION | 0 | 0 | 0 |
|  |  | KEGG_HUNTINGTONS_DISEASE | BDNF | CREBBP CREB1 HIP1  GNAQ CLTC CREB3L2 CLTCL1 PPARG COX6C | EP300 SDHC SDHD SDHB TP53 |
|  |  | KEGG_PYRUVATE_METABOLISM | 0 | 0 | 0 |
|  | GO | GOBP_CHROMOSOME_SEGREGATION | 0 | EML4 STIL NUMA1 BCL7A TPR KNL1 TOP1 TTL CCNB1IP1 | RB1 SMARCB1 PTEN BUB1B BRCA1 MLH1 SMARCA4 BRIP1 APC FANCD2 CHEK2 ERCC2  ATM |
|  |  | GOBP_REGULATION_OF_CHROMOSOM E_ORGANIZATION | 0 | TFPT NUMA1 BCL7A TAL1 MYC HNRNPA2B1 TPR CTNNB1 KNL1  PML | RB1 SMARCB1 APC ERCC4 BUB1B ATM NBN TP53 SMARCA4 |
|  |  | GOBP_RIBONUCLEOPROTEIN_COMPL EX_BIOGENESIS | SBDS | HSP90AA1 NPM1  HSP90AB1 CLP1 PSIP1 DDX10 | ERCC2 SBDS ATM TSC1 DICER1 |
|  |  | GOCC_MITOCHONDRIAL_PROTEIN_C  ONTAINING_COMPLEX | 0 | COX6C | SDHC SDHD SDHB |
|  |  | GOCC_ORGANELLE_INNER_MEMBRAN E |  | COX6C | SDHC SDHD SDHB |
