# Supplementary material for: Hypoxia promotes progression of cervical cancer by modulating the ATXN3-enhanced P53 stability or STAT5 phosphorylation
Source: Cell Death Discov. 2026 Jan 8;12:4. doi: 10.1038/s41420-025-02822-0 (PMC12783129; doi:10.1038/s41420-025-02822-0)
Supplement: Supplementary file 6 — Supplementary Table 6 [file 41420_2025_2822_MOESM6_ESM.docx]

| Supplementary Table 6：Binding Energy for ATXN3 Complexes with p-JAK3, STAT5, and p-STAT5. (kcal/mol) | | | | | |
| --- | --- | --- | --- | --- | --- |
| 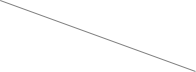 Receptor: Target/protein/PDB/small molecule/CID/CAS | | pJAK3-980 | STAT5 | pSTAT5-694 | -10 |
| Ligand: Protein (polypeptide)/PDB | | 3PJC | 7UBT | 7UBT | -9 |
| ATXN3 | 4YS9 | -11.5 | -8.9 | -9.2 | -8 |
|  |  |  |  |  | -7 |
|  |  |  |  |  | -6 |
|  |  |  |  |  | -5 |
|  |  |  |  |  | -4 |
|  |  |  |  |  | -3 |
|  |  |  |  |  | -2 |
|  |  |  |  |  | -1 |
|  |  |  |  |  | 0 |
