# Supplementary figures and images for: Hypoxia promotes progression of cervical cancer by modulating the ATXN3-enhanced P53 stability or STAT5 phosphorylation
Source: Cell Death Discov. 2026 Jan 8;12:4. doi: 10.1038/s41420-025-02822-0 (PMC12783129; doi:10.1038/s41420-025-02822-0)

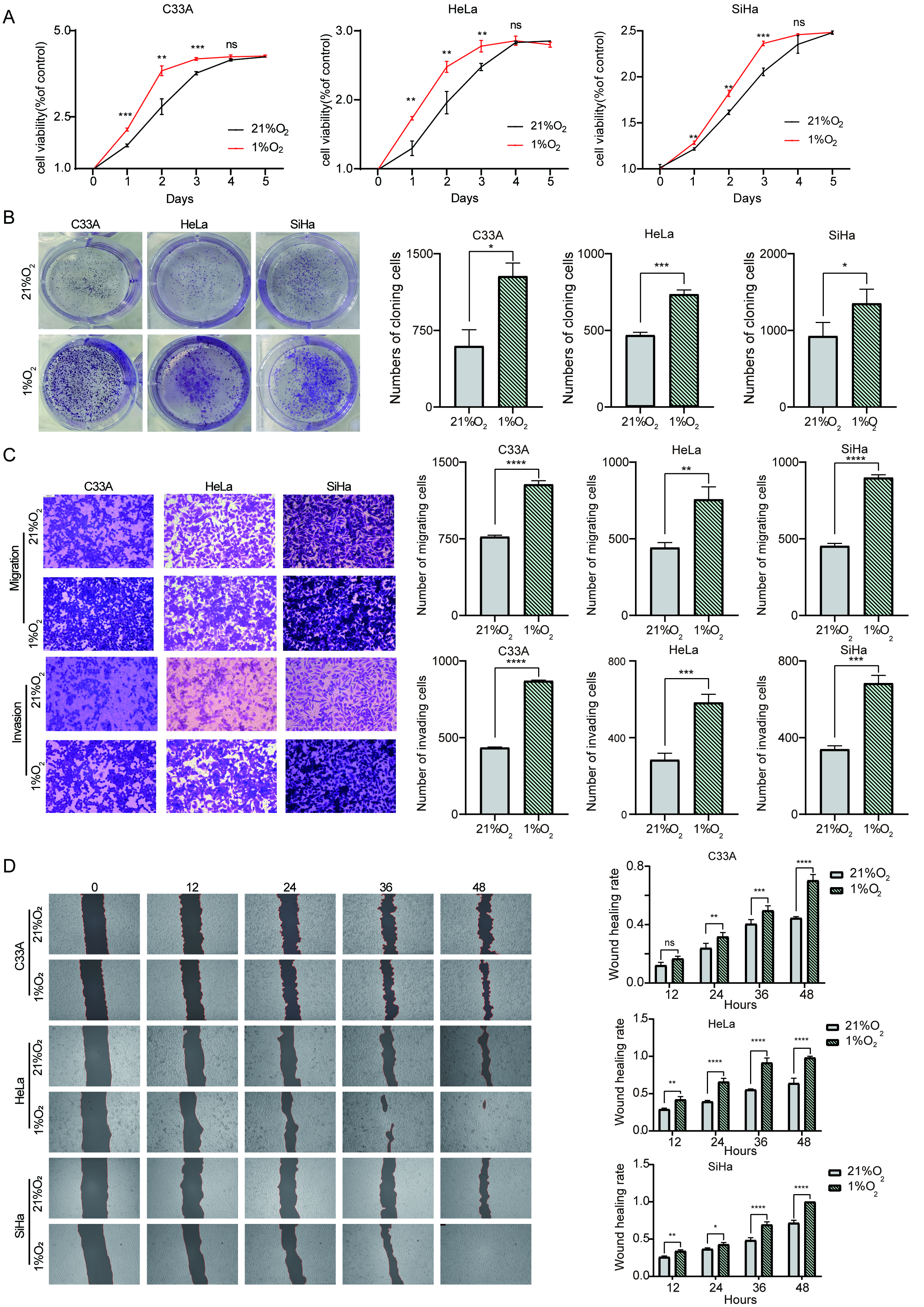

Supplement: Supplementary file 7 — Supplementary Figure 1 [file 41420_2025_2822_MOESM7_ESM.tif]

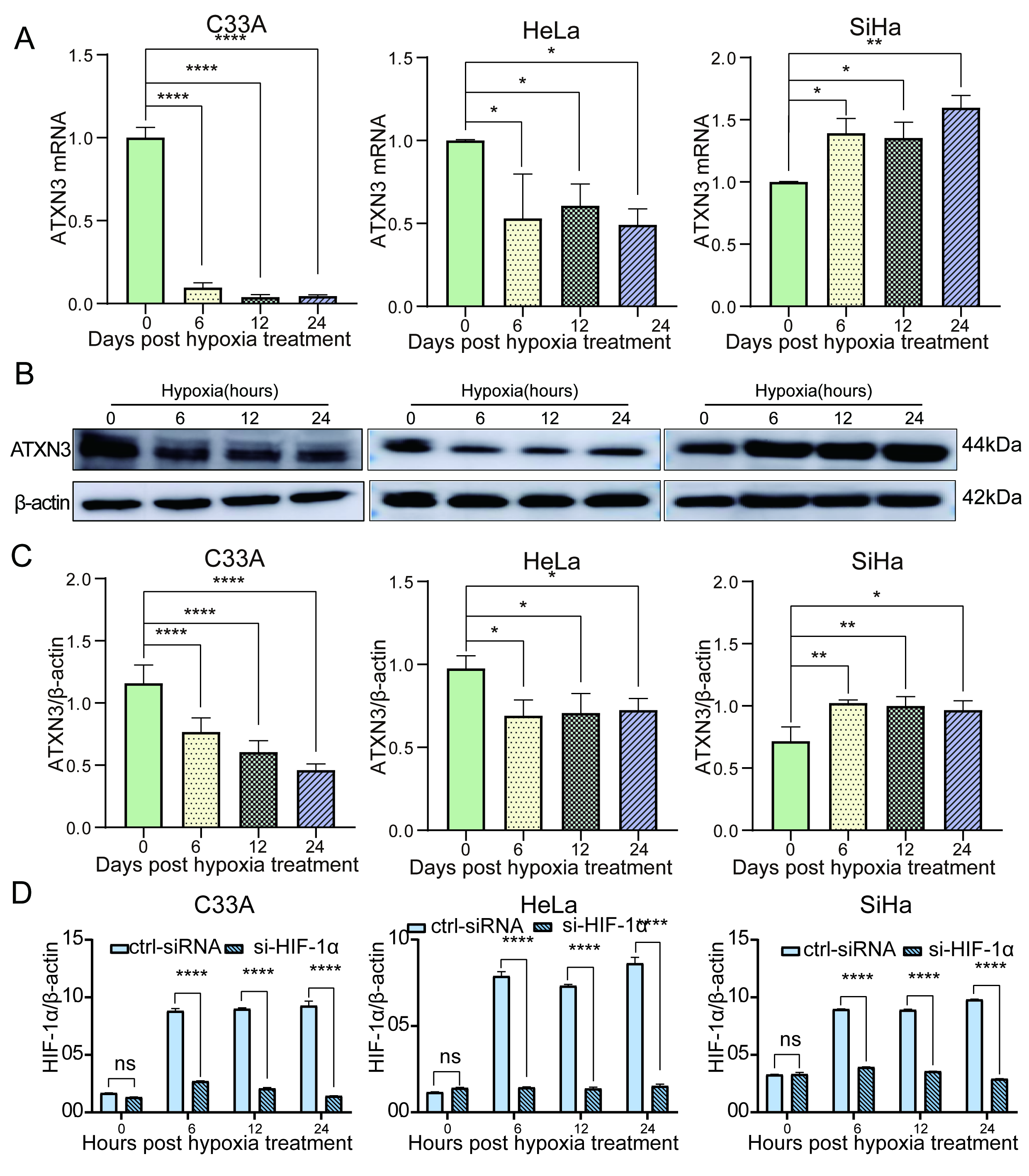

Supplement: Supplementary file 8 — Supplementary Figure 2 [file 41420_2025_2822_MOESM8_ESM.tif]

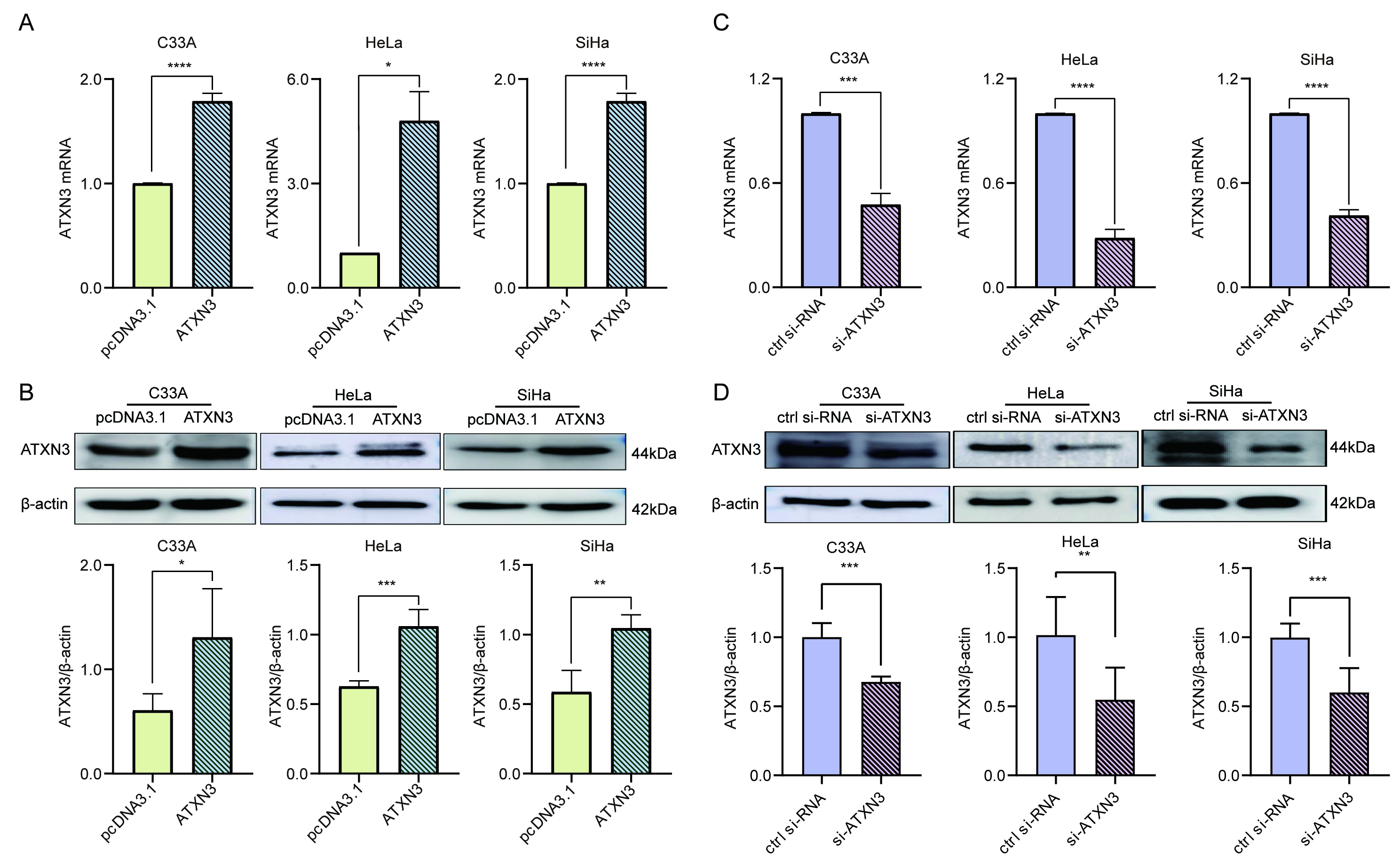

Supplement: Supplementary file 9 — Supplementary Figure 3 [file 41420_2025_2822_MOESM9_ESM.tif]

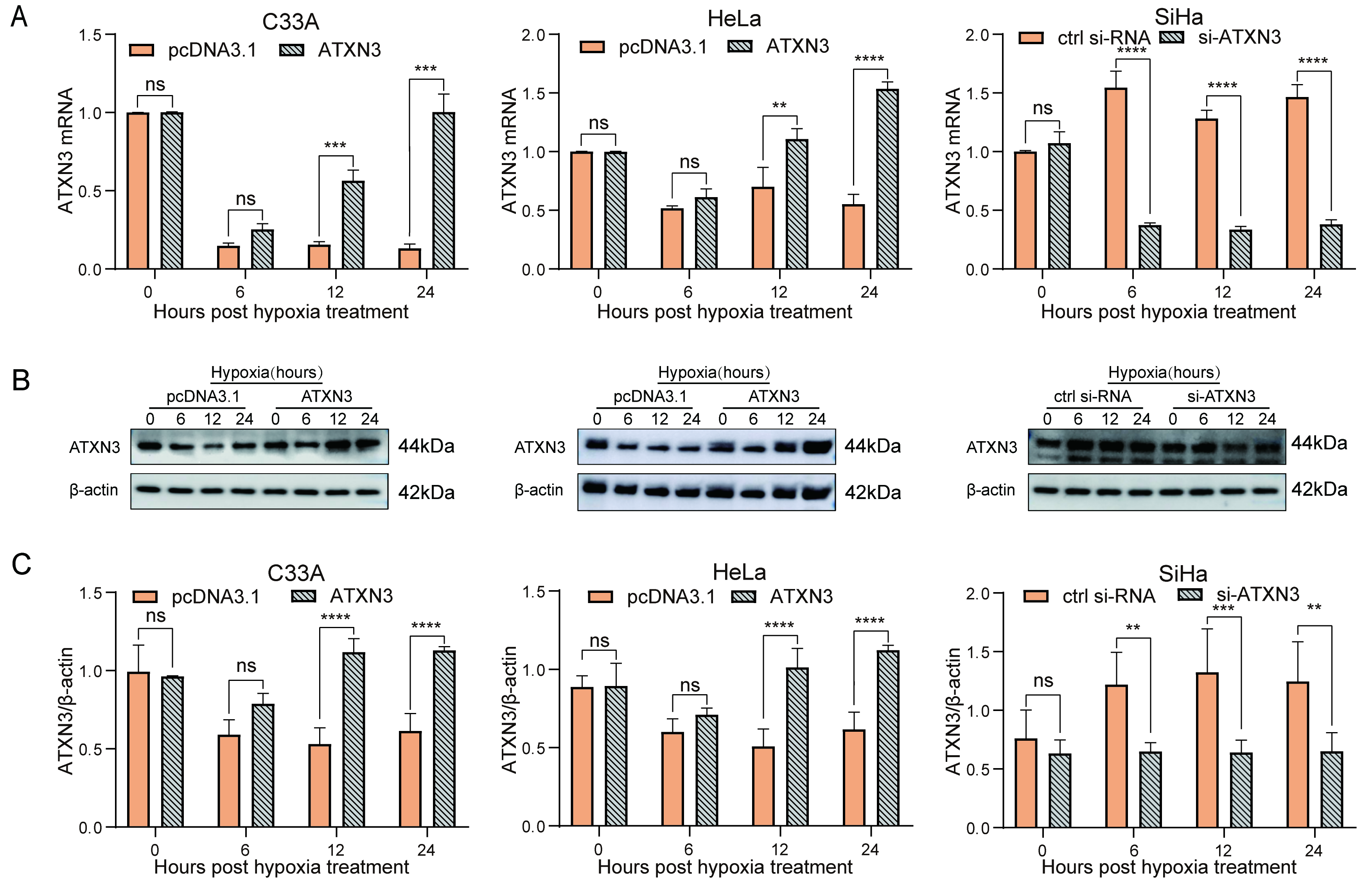

Supplement: Supplementary file 10 — Supplementary Figure 4 [file 41420_2025_2822_MOESM10_ESM.tif]

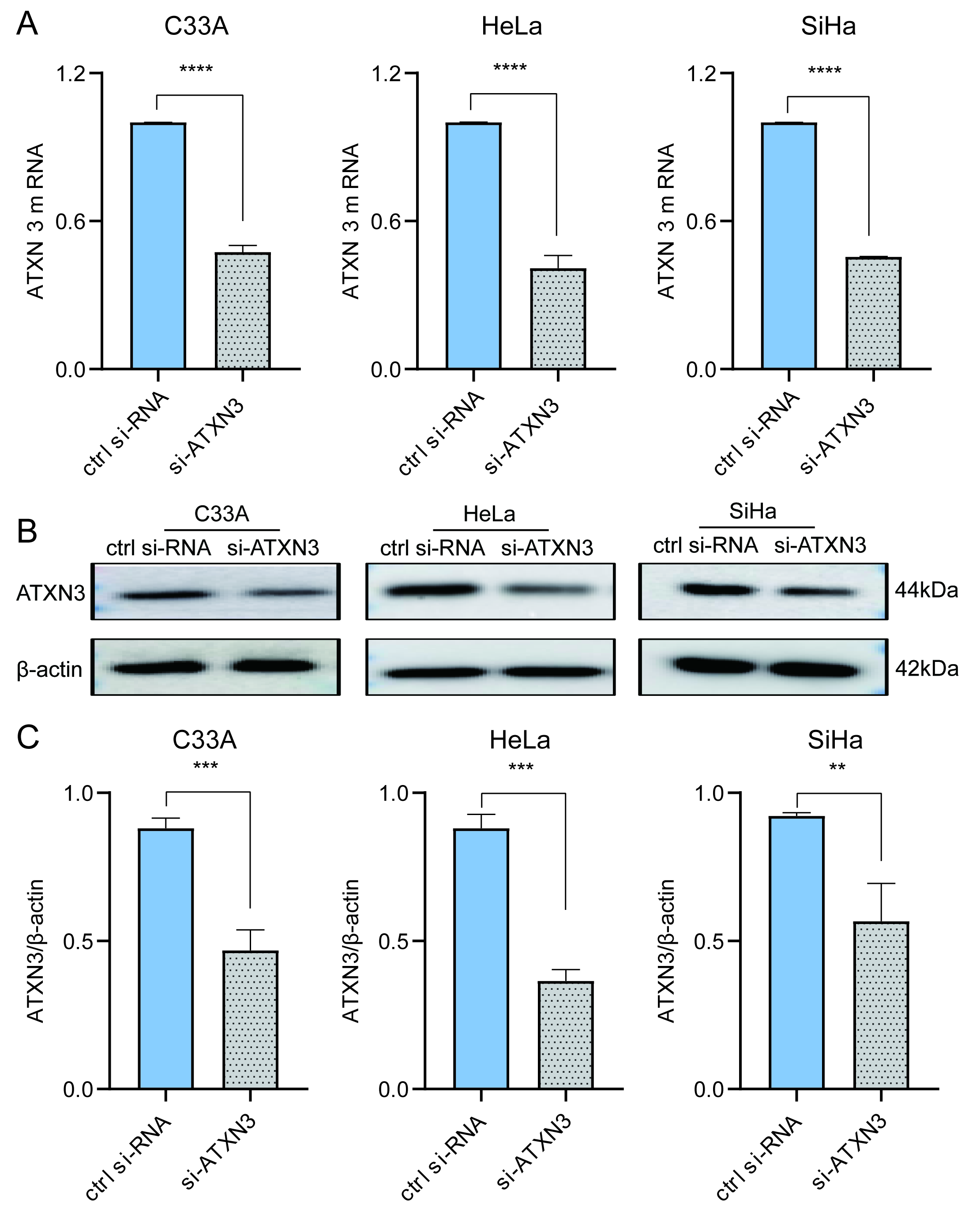

Supplement: Supplementary file 11 — Supplementary Figure 5 [file 41420_2025_2822_MOESM11_ESM.tif]

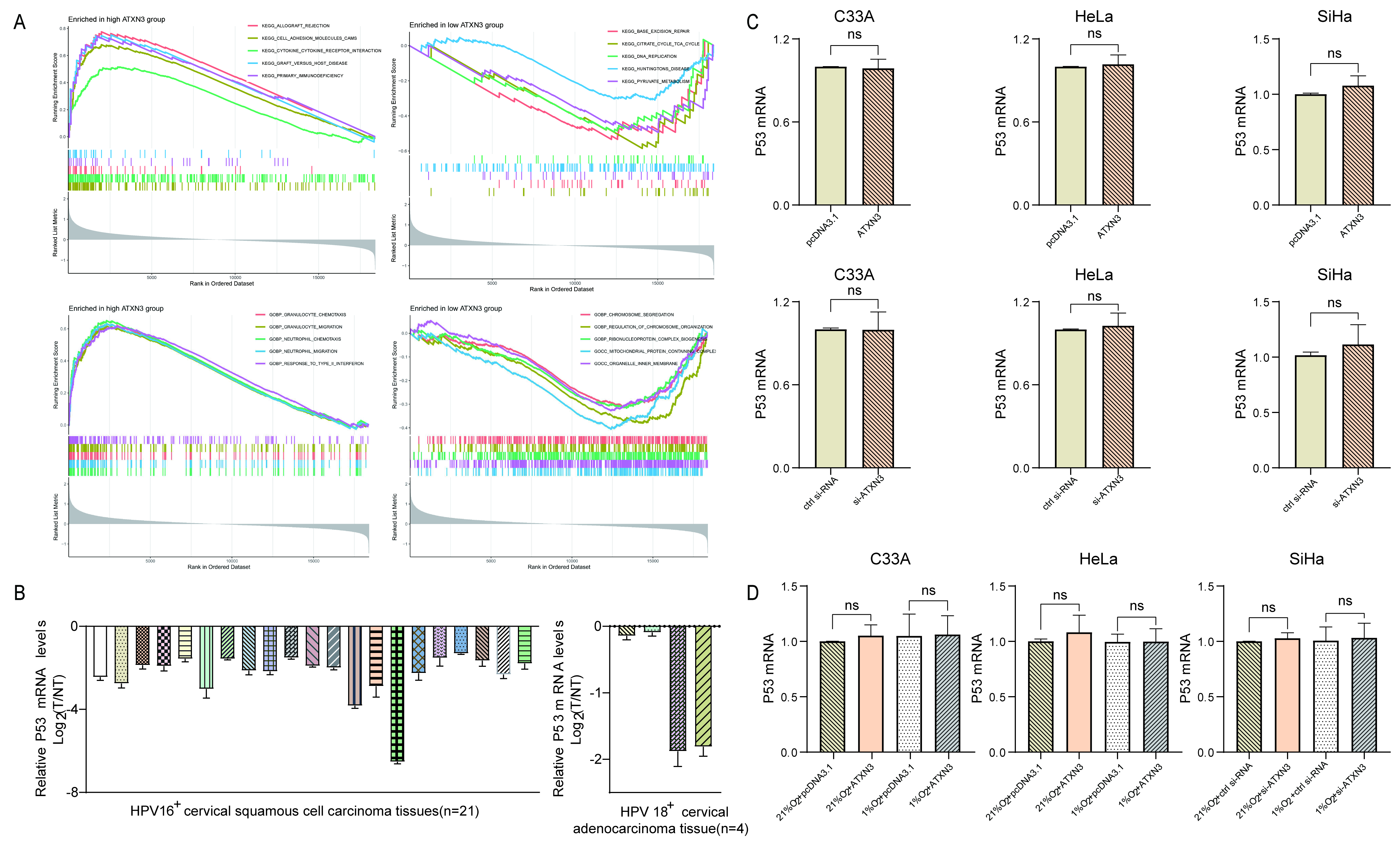

Supplement: Supplementary file 12 — Supplementary Figure 6 [file 41420_2025_2822_MOESM12_ESM.tif]

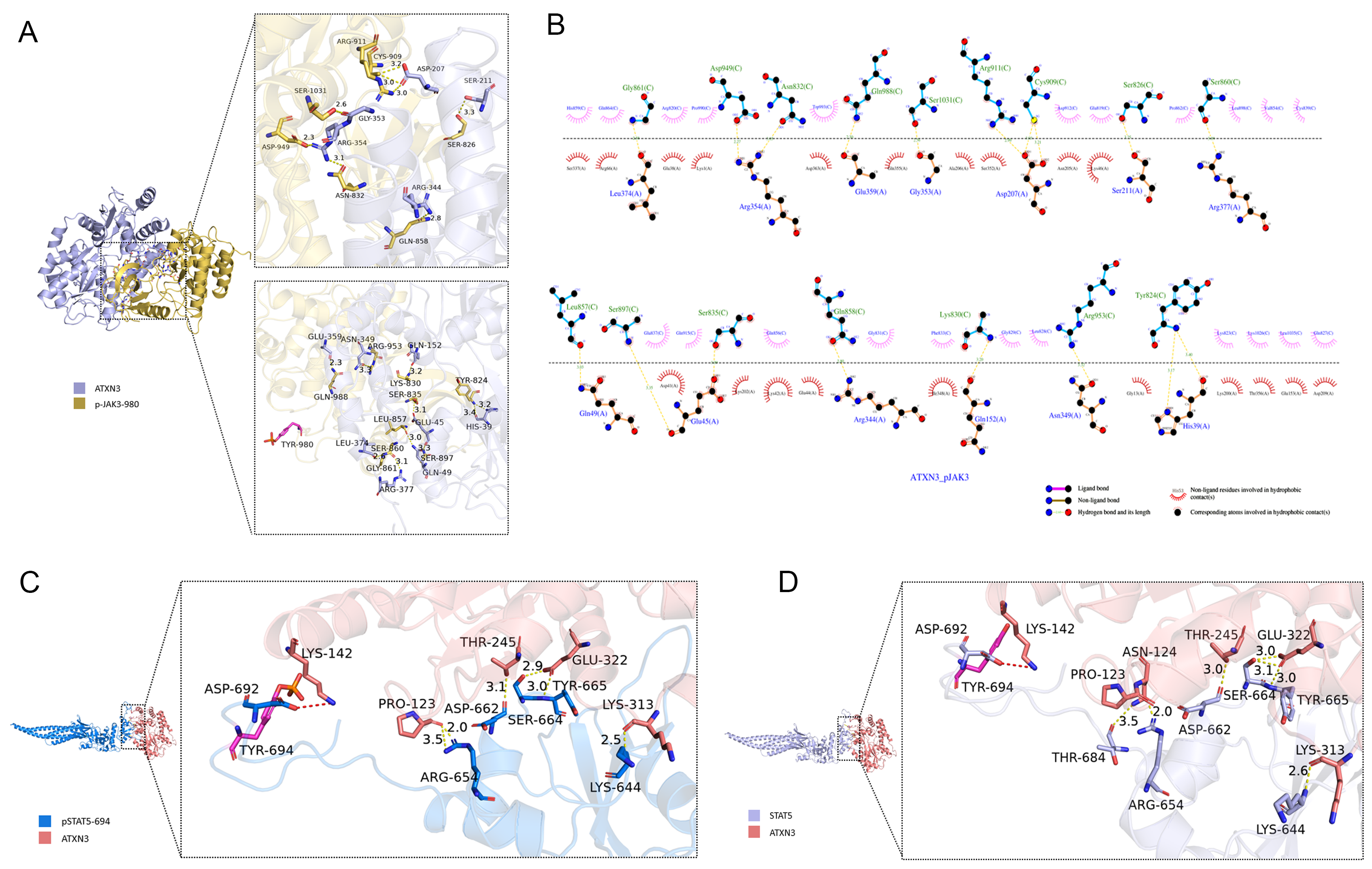

Supplement: Supplementary file 13 — Supplementary Figure 7 [file 41420_2025_2822_MOESM13_ESM.tif]

The original, unprocessed images of the nude mice and tumors are provided below:

C33A

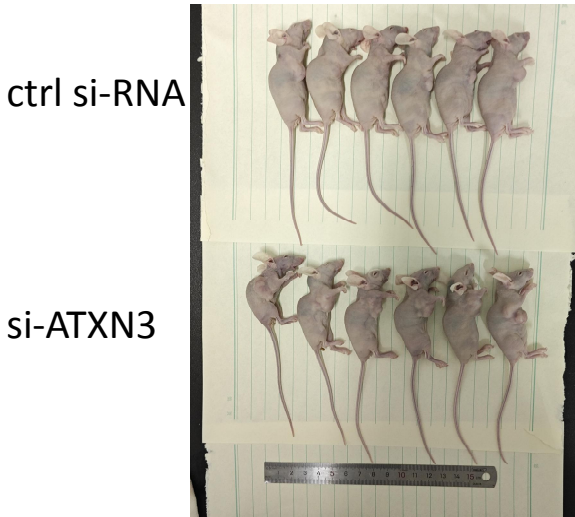

HeLa

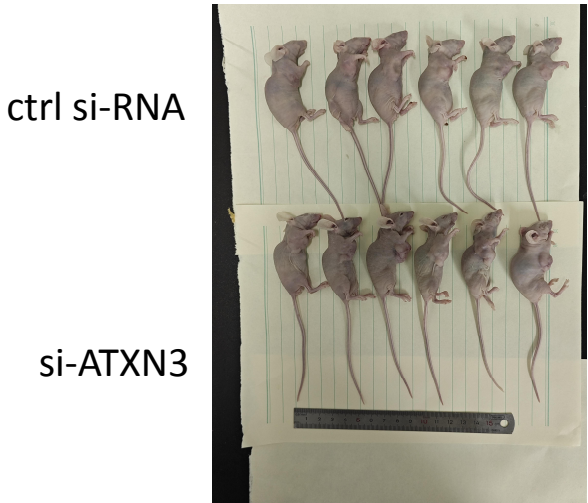

SiHa

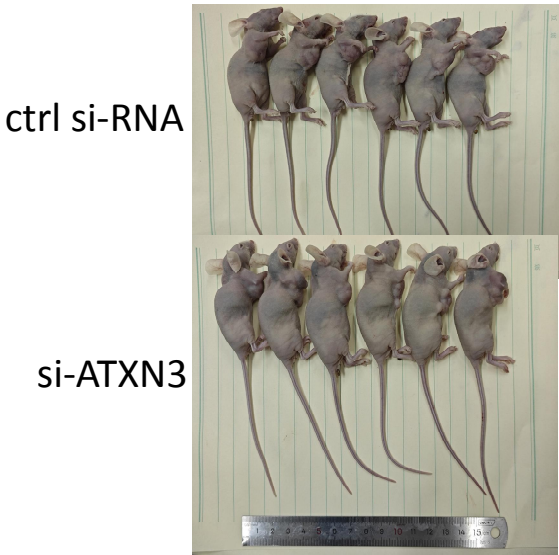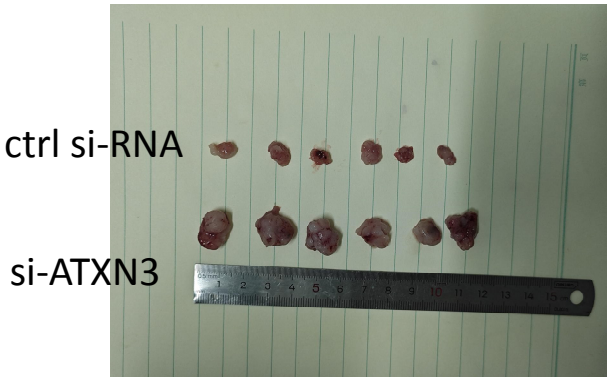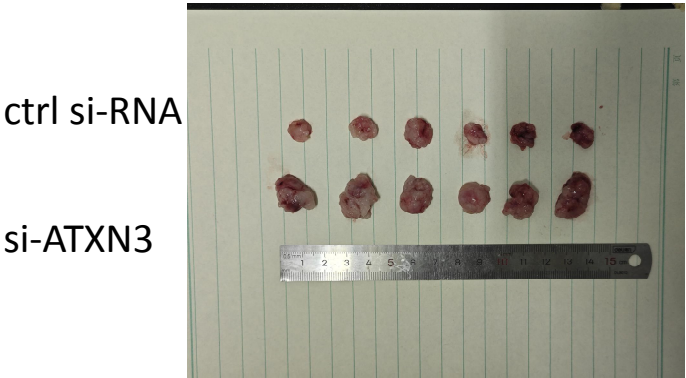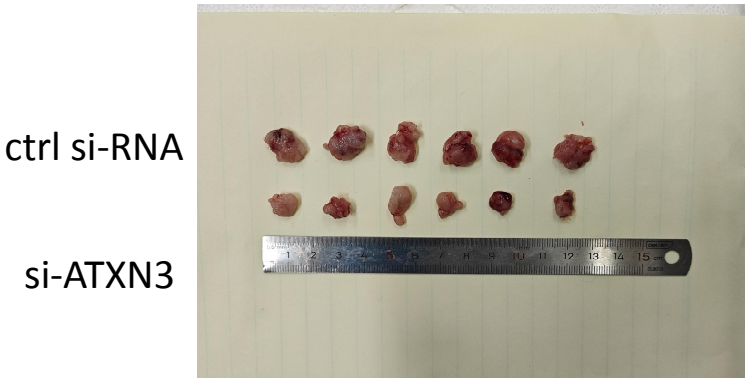

Supplement: Supplementary file 16 — Supplementary File [file 41420_2025_2822_MOESM16_ESM.pdf]
