# Supplementary material for: Hypoxia promotes progression of cervical cancer by modulating the ATXN3-enhanced P53 stability or STAT5 phosphorylation
Source: Cell Death Discov. 2026 Jan 8;12:4. doi: 10.1038/s41420-025-02822-0 (PMC12783129; doi:10.1038/s41420-025-02822-0)
Supplement: Supplementary file 14 — Supplementary table legend [file 41420_2025_2822_MOESM14_ESM.docx]

Supplementary Table 1: Recognition sequences of HIF-1α obtained from the JASPAR database.

Supplementary Table 2: ATXN3 promoter—HIF-1α target sequences.

Supplementary Table 3: Human sequence of ATXN3 promoter.

Supplementary Table 4: GSEA analysis of ATXN3.

Supplementary Table 5: cancer-related genes were systematically annotated and classified within significantly enriched pathways.

Supplementary Table 6：Binding Energy for ATXN3 Complexes with p-JAK3, STAT5, and p-STAT5.
