# Supplementary material for: Hypoxia promotes progression of cervical cancer by modulating the ATXN3-enhanced P53 stability or STAT5 phosphorylation
Source: Cell Death Discov. 2026 Jan 8;12:4. doi: 10.1038/s41420-025-02822-0 (PMC12783129; doi:10.1038/s41420-025-02822-0)
Supplement: Supplementary file 15 — Supplementary figure legend [file 41420_2025_2822_MOESM15_ESM.docx]

Supplementary Figure 1. Hypoxia promotes proliferation, migration and invasion of cervical cancer cells. A. MTS assay showing the effect of hypoxia on cervical cancer cell proliferation. B. Colony formation assay demonstrating the impact of hypoxia on cervical cancer cell proliferation. C. Transwell assay evaluating the influence of hypoxia on cervical cancer cell migration and invasion. D. Wound healing assay assessing hypoxia-induced cervical cancer cell migration.

Supplementary Figure 2. Effecience of short-term hypoxia on ATXN3 in cervical cancer cells. A. Short-term hypoxia affects ATXN3 mRNA expression in cervical cancer cells.B-C. Short-term hypoxia affects ATXN3 protein expression in cervical cancer cells. D. Statistical graph of the effect of HIF-1α knockdown on ATXN3 protein.

Supplementary Figure 3. Effects of ATXN3 overexpression and knockdown. A-B. Validation of ATXN3 overexpression efficiency.C-D. Validation of ATXN3 knockout efficiency.

Supplementary Figure 4. ATXN3 intervention rescues cervical cancer cells under hypoxia. A ATXN3 intervention rescues ATXN3 mRNA expression in cervical cancer cells under hypoxic conditions. B-C ATXN3 intervention rescues ATXN3 protein in cervical cancer cells under hypoxic conditions.

Supplementary Figure 5.Effects of ATXN3 knockdown. A. Validation of ATXN3 mRNA knockout efficiency. B-C. Validation of ATXN3 protein knockout efficiency.

Supplementary Figure 6. GSEA analysis of ATXN3 and detection of P53 mRNA levels. A GSEA analysis of ATXN3. B. mRNA expression of P53 in cervical cancer tissues. C. Effect of ATXN3 intervention on P53 mRNA expression in cervical cancer cells. D. Effect of hypoxia combined with ATXN3 rescue on P53 mRNA expression.

Supplementary Figure 7.Molecular docking of ATXN3. A. Molecular docking between ATXN3 and p-JAK3. B. Bond interactions between ATXN3 and p-JAK3. C. Molecular docking between ATXN3 and p-STAT5. D. Molecular docking between ATXN3 and STAT5.
