# Supplementary material for: Hypoxia promotes progression of cervical cancer by modulating the ATXN3-enhanced P53 stability or STAT5 phosphorylation
Source: Cell Death Discov. 2026 Jan 8;12:4. doi: 10.1038/s41420-025-02822-0 (PMC12783129; doi:10.1038/s41420-025-02822-0)
Supplement: Supplementary file 17 — Original western blots [file 41420_2025_2822_MOESM17_ESM.pdf]

Figure 2B

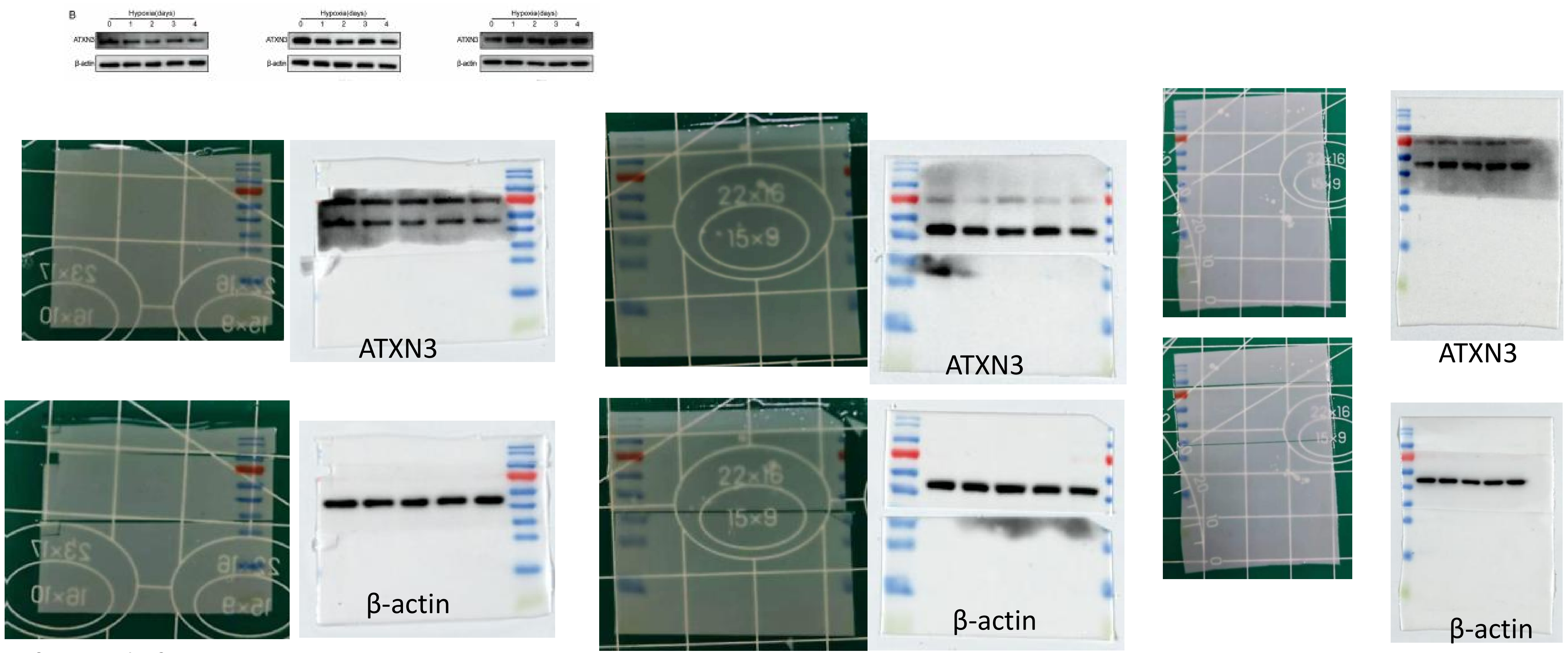

Before and after cropping

Before and after cropping

Before and after cropping

Figure 2E

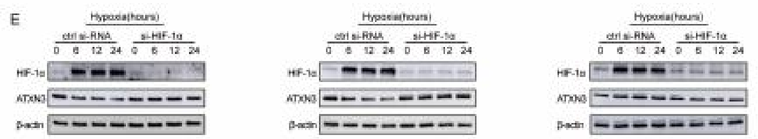

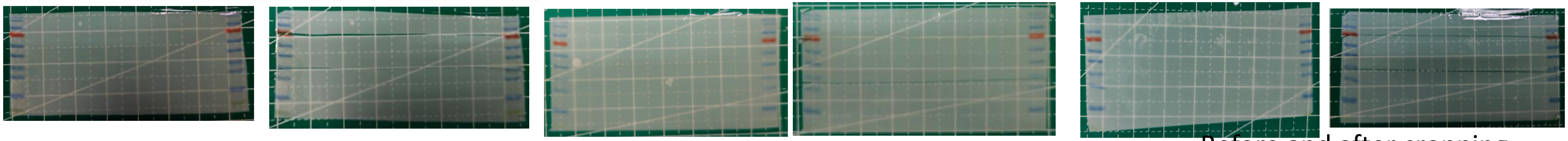

Before and after cropping

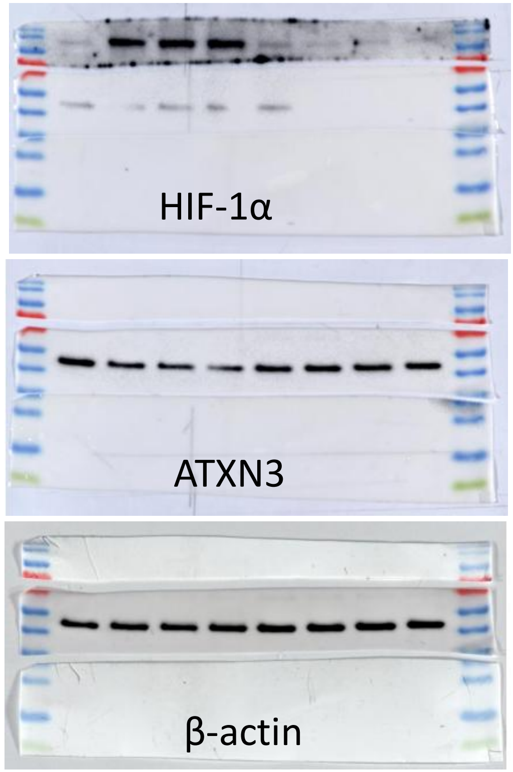

Before and after cropping

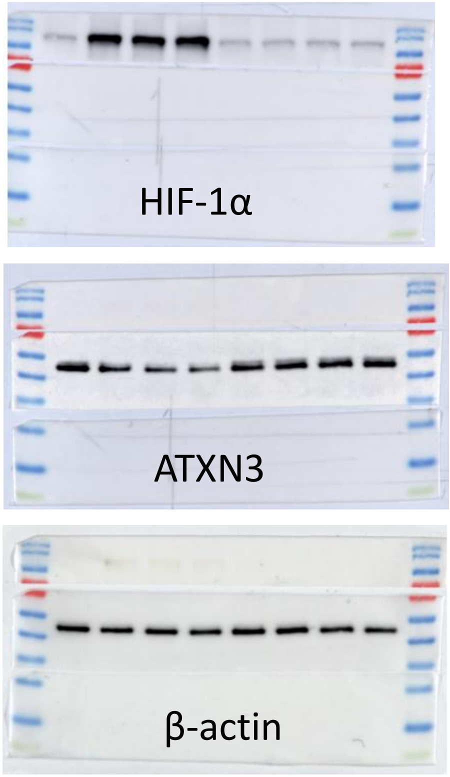

Before and after cropping

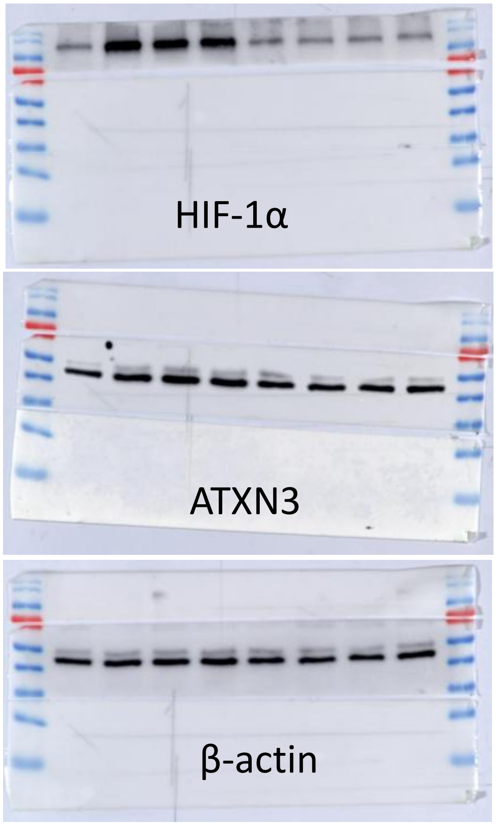

Supplementary Figure 2B

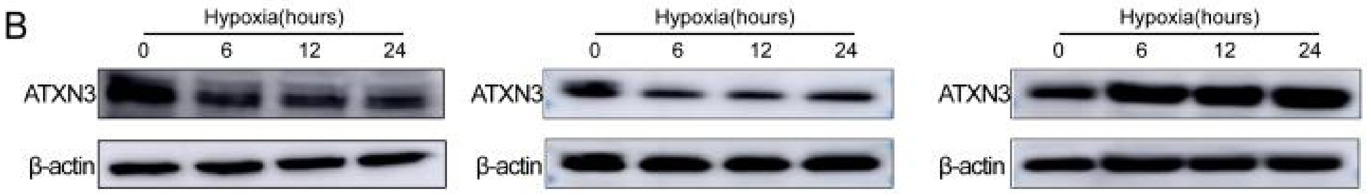

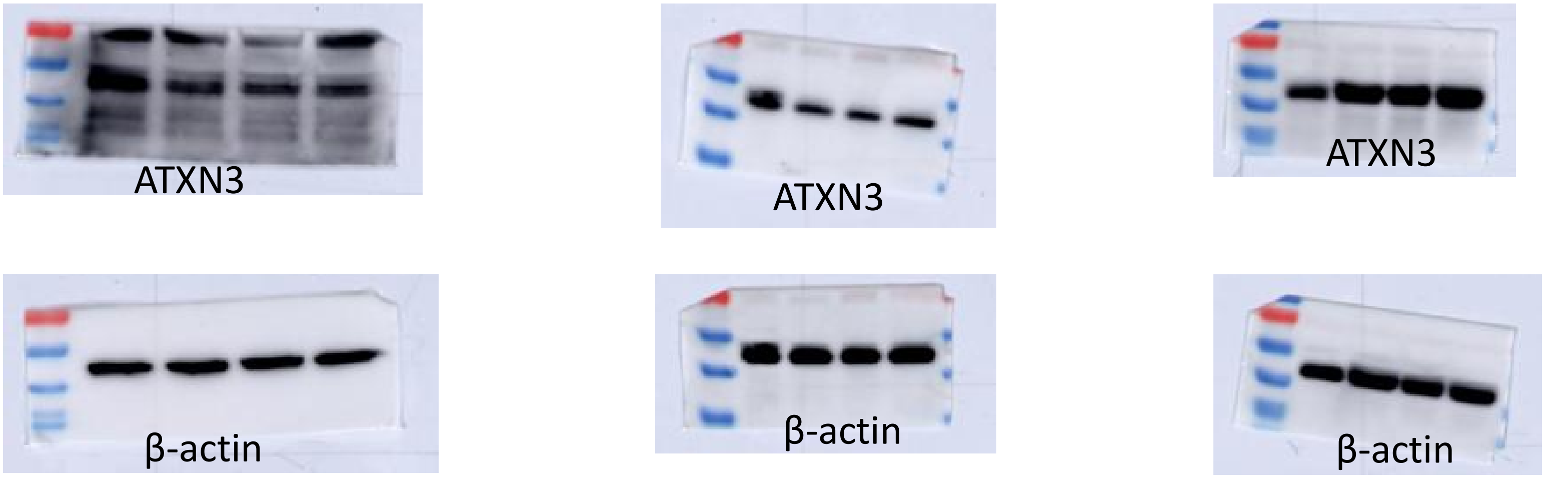

Supplementary Figure 3B and D

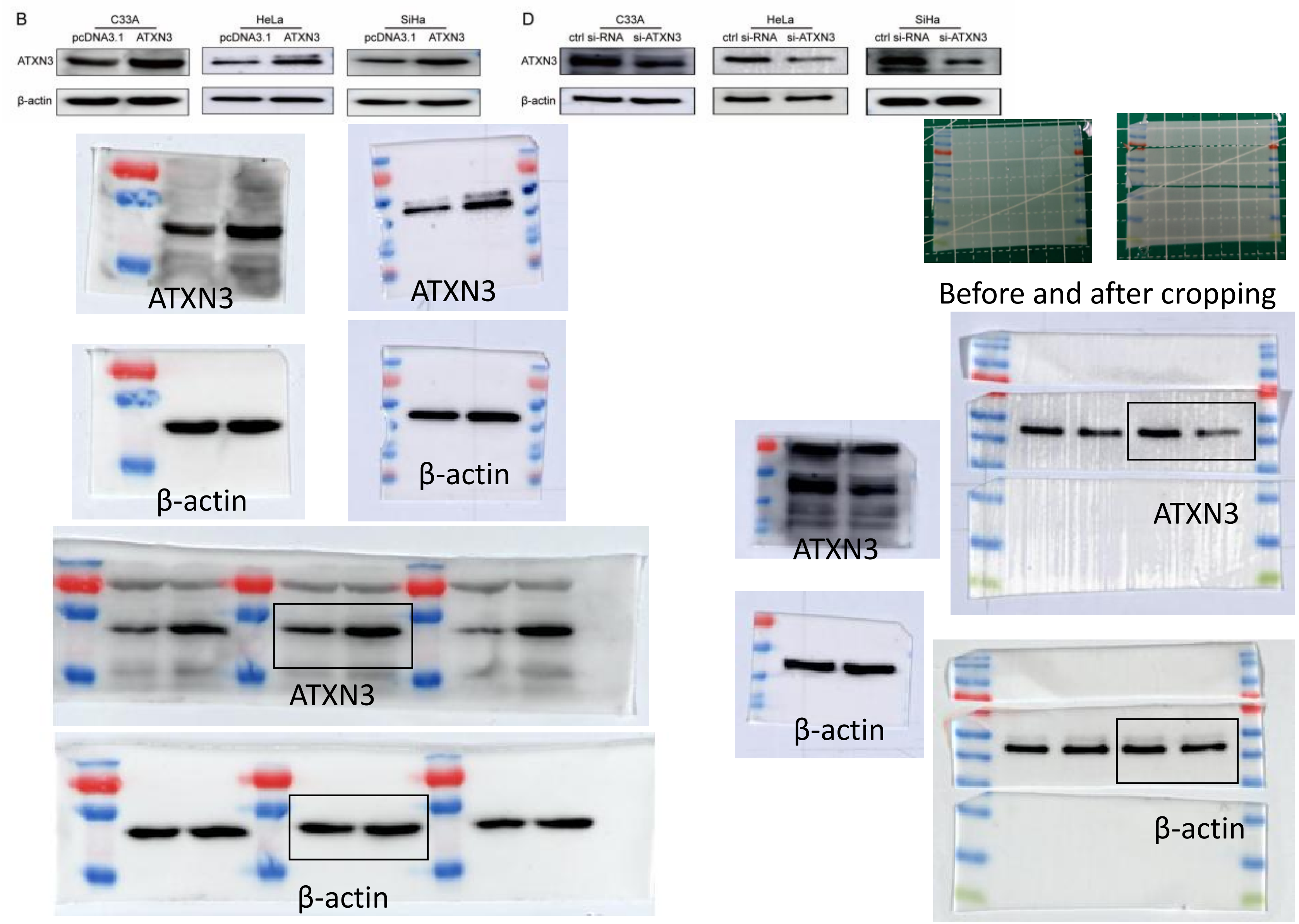

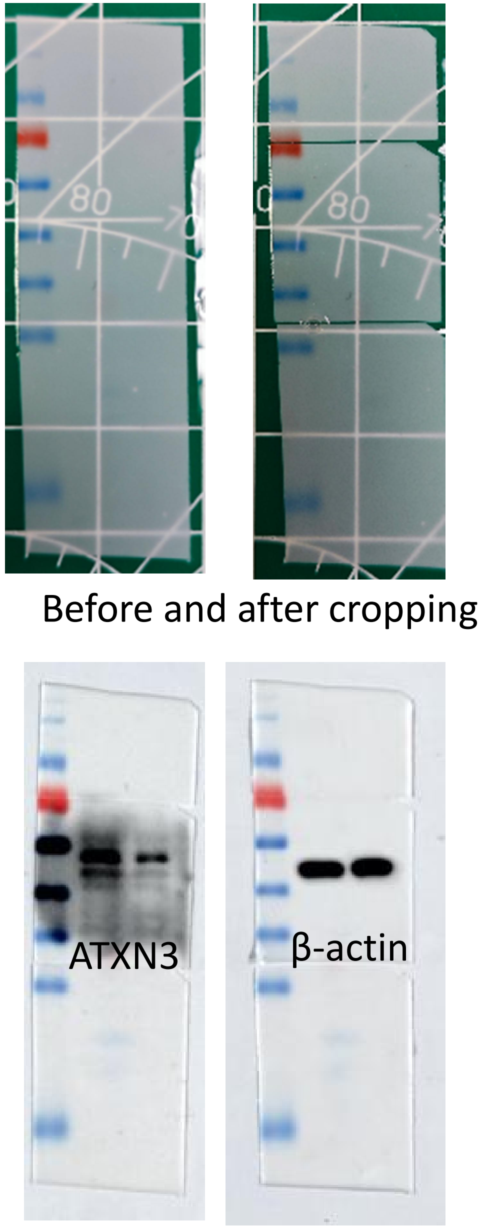

Supplementary Figure 4B

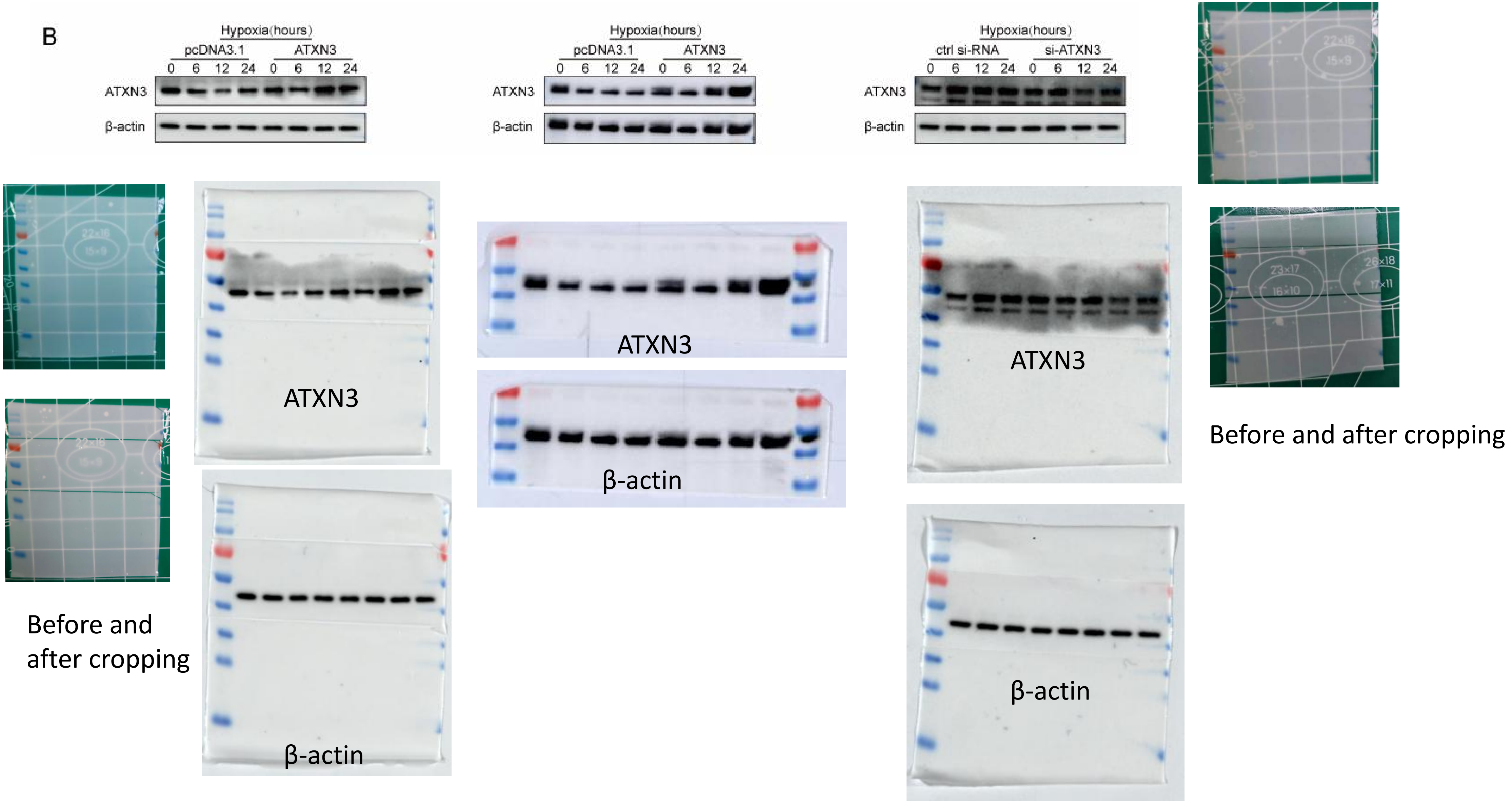

Supplementary Figure 5B

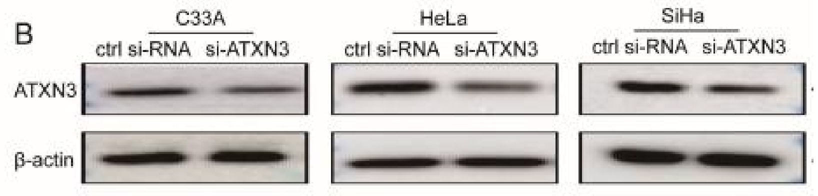

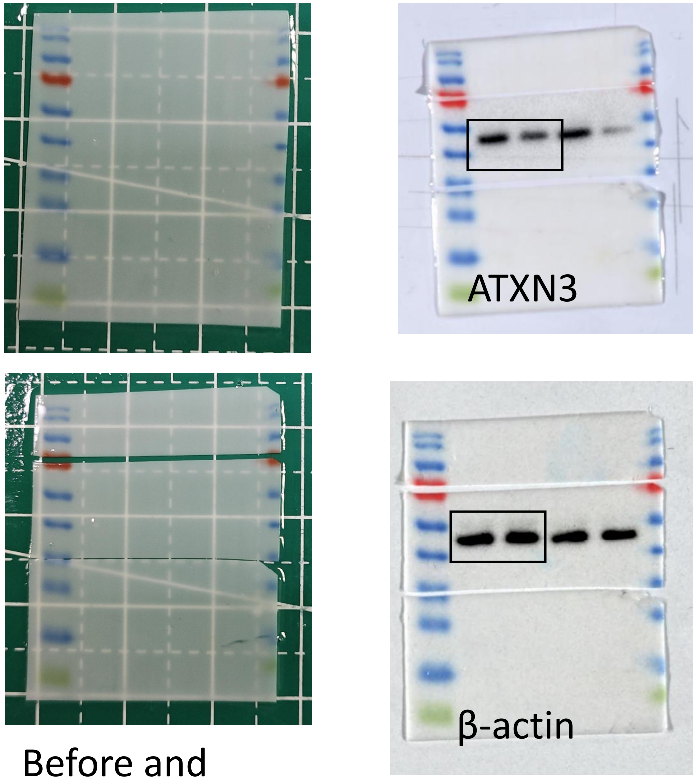

Before and after cropping

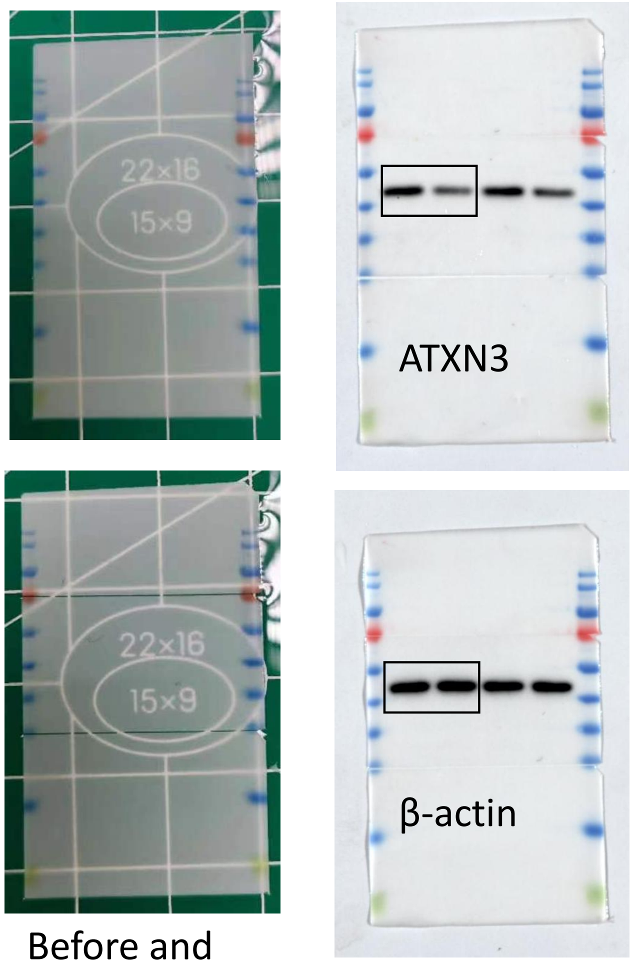

Before and after cropping

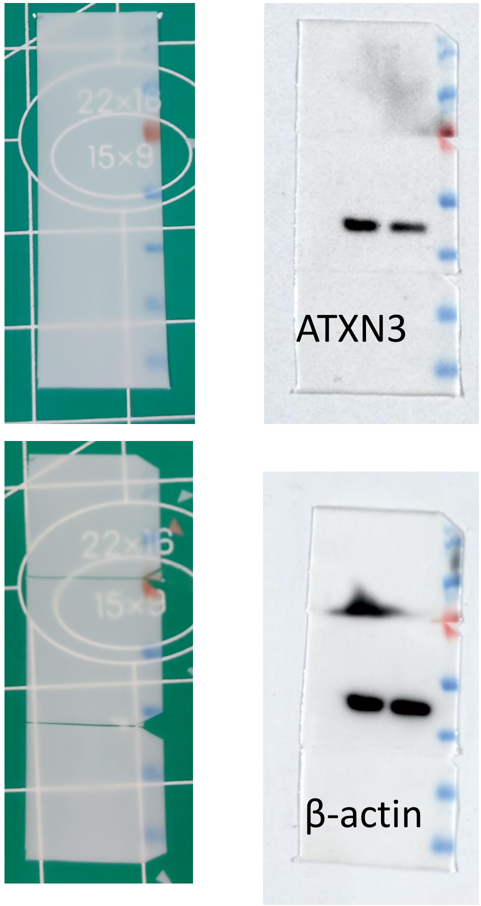

Before and after cropping

Figure 7F

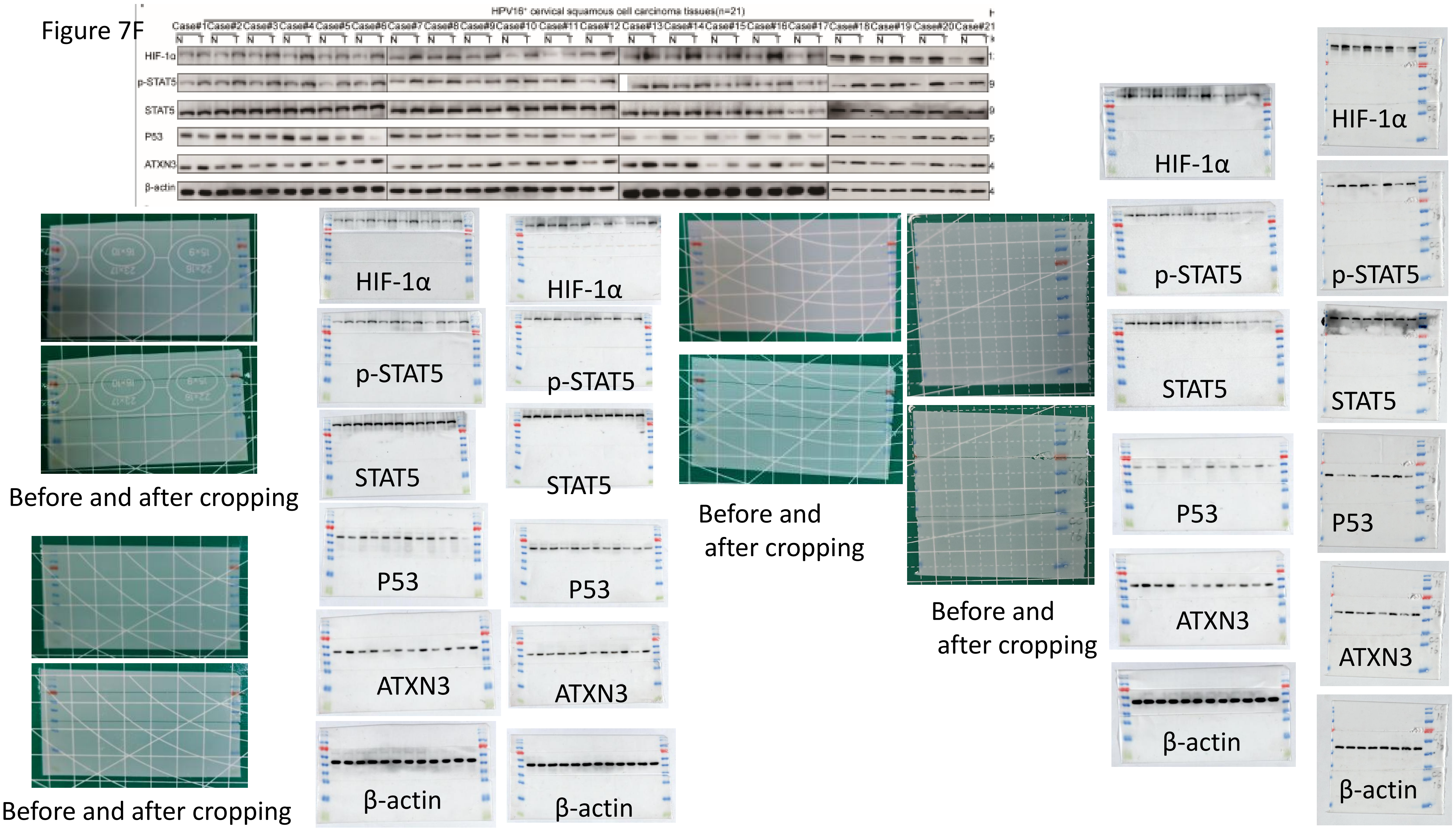
HPV16⁺ cervical squamous cell carcinoma tissues(n=21)
HIF-1α
p-STAT5
STAT5
P53
ATXN3
β-actin
Before and after cropping
HIF-1α
p-STAT5
STAT5
P53
ATXN3
β-actin
HIF-1α
p-STAT5
STAT5
P53
ATXN3
β-actin
Before and after cropping
Before and after cropping
Before and after cropping
HIF-1α
p-STAT5
STAT5
P53
ATXN3
β-actin
HIF-1α
p-STAT5
STAT5
P53
ATXN3
β-actin

Figure 7I

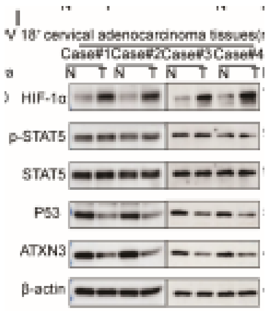

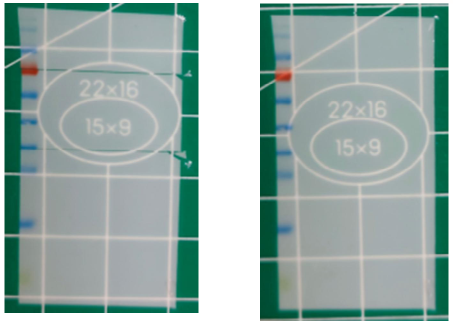

Before and after cropping

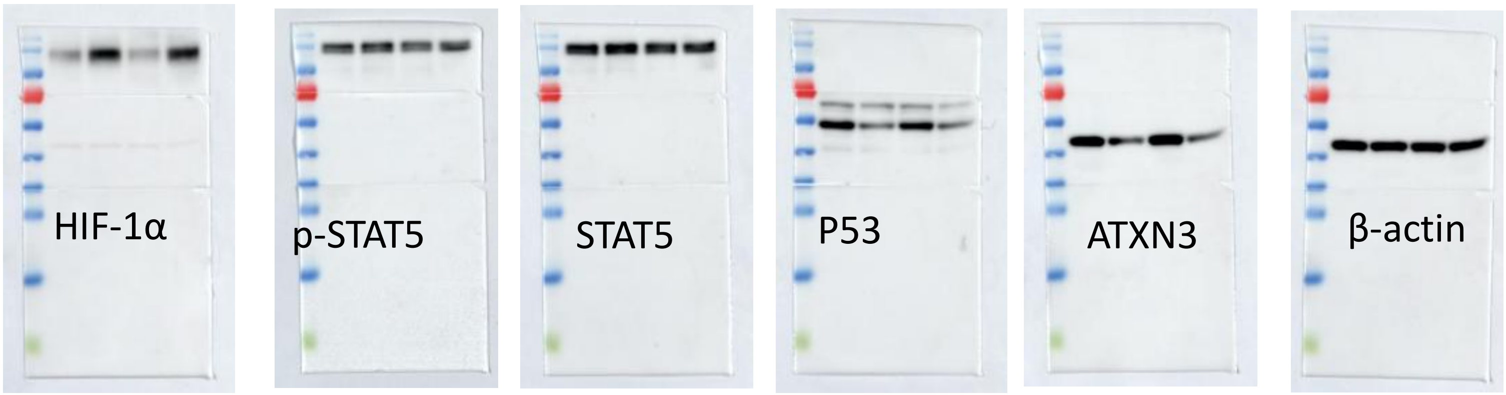

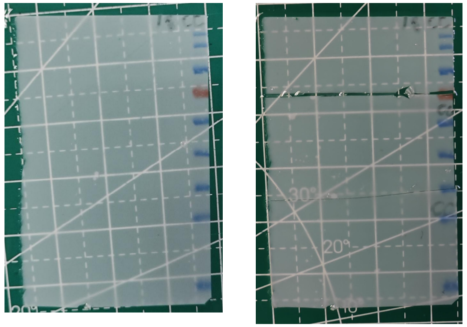

Before and after cropping

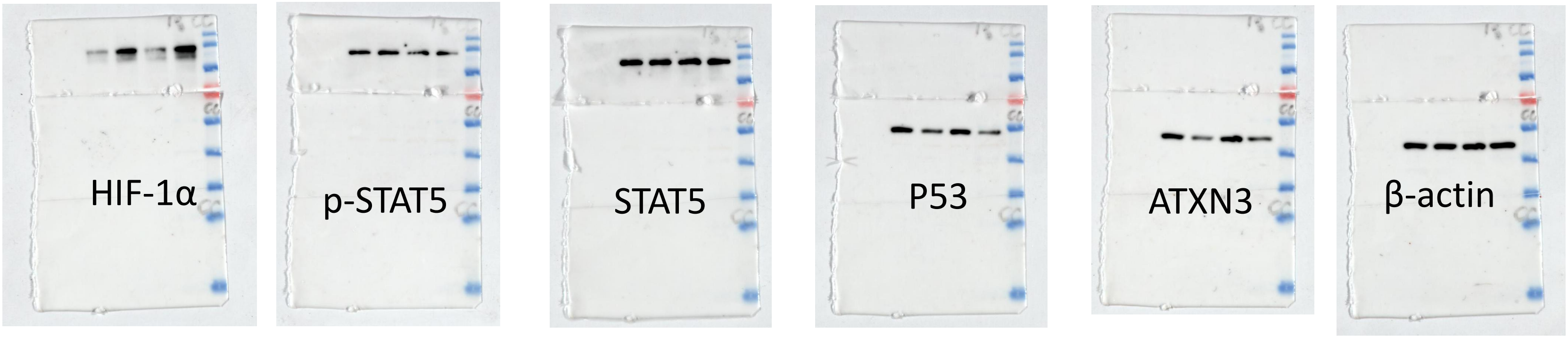

Figure 8A

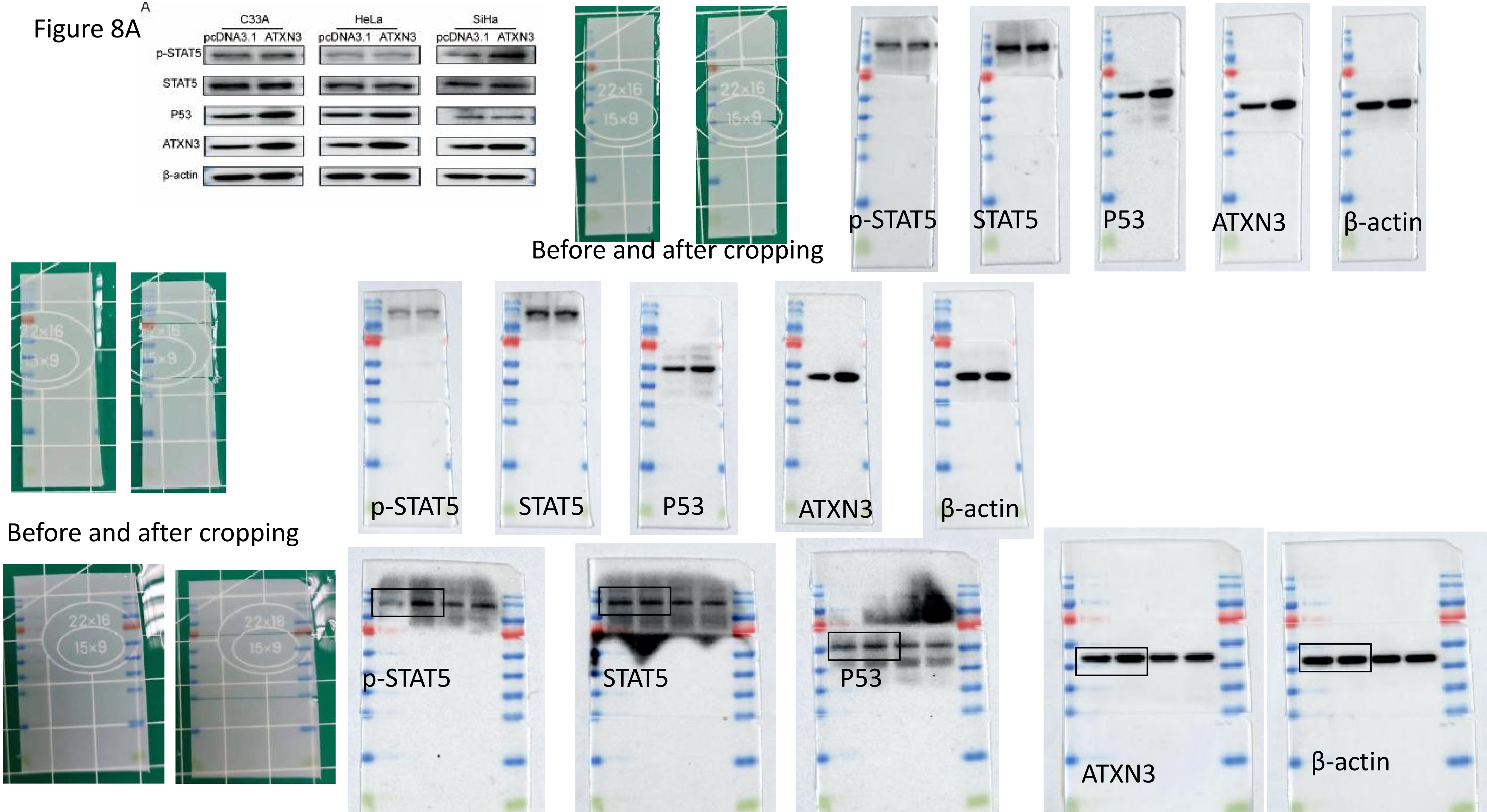

Before and after cropping

Figure 8D

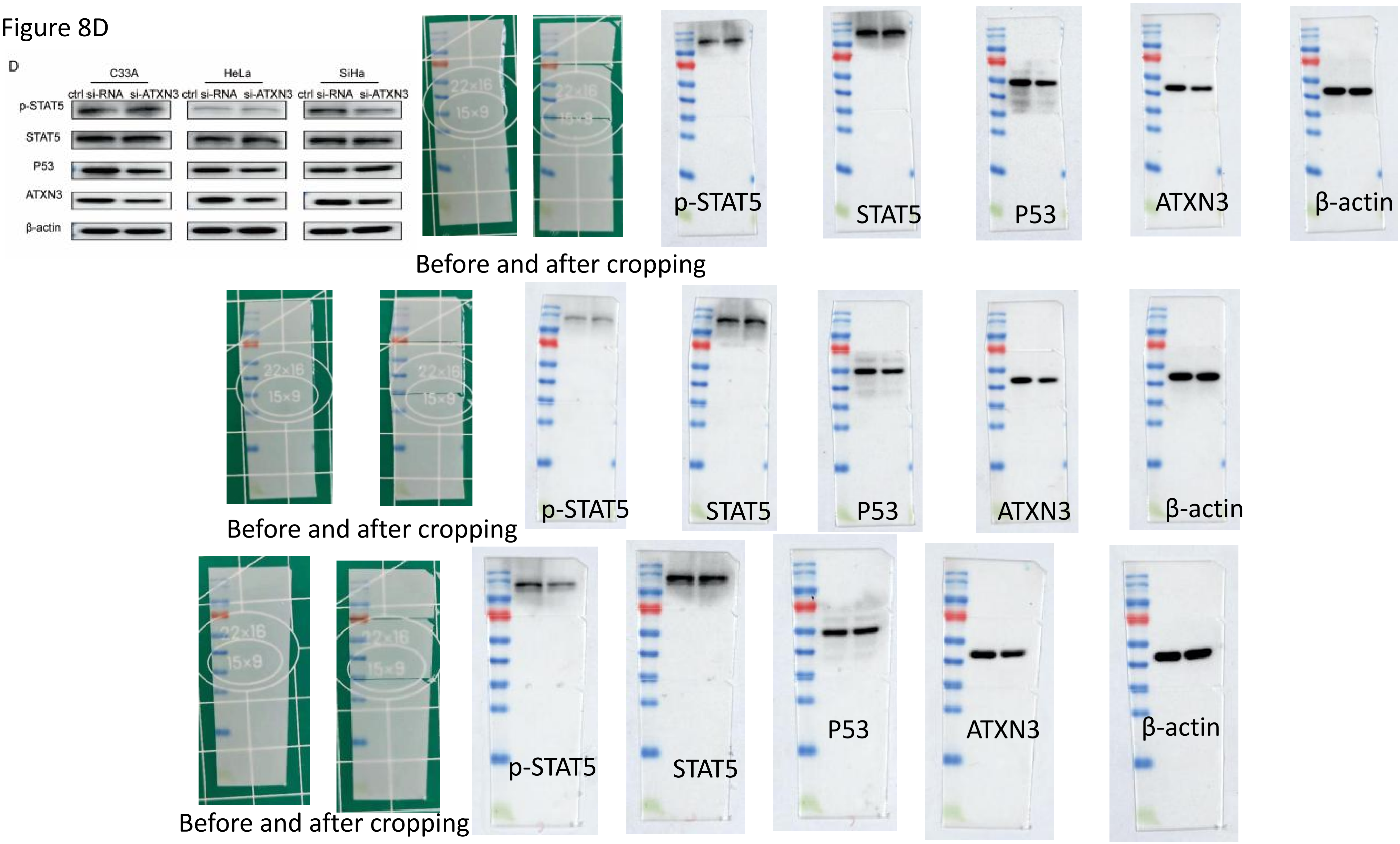
D
C33A
HeLa
SiHa
ctrl si-RNA
si-ATXN3
p-STAT5
STAT5
P53
ATXN3
β-actin
Before and after cropping
p-STAT5
STAT5
P53
ATXN3
β-actin
Before and after cropping
p-STAT5
STAT5
P53
ATXN3
β-actin
Before and after cropping
p-STAT5
STAT5
P53
ATXN3
β-actin

Figure 8G

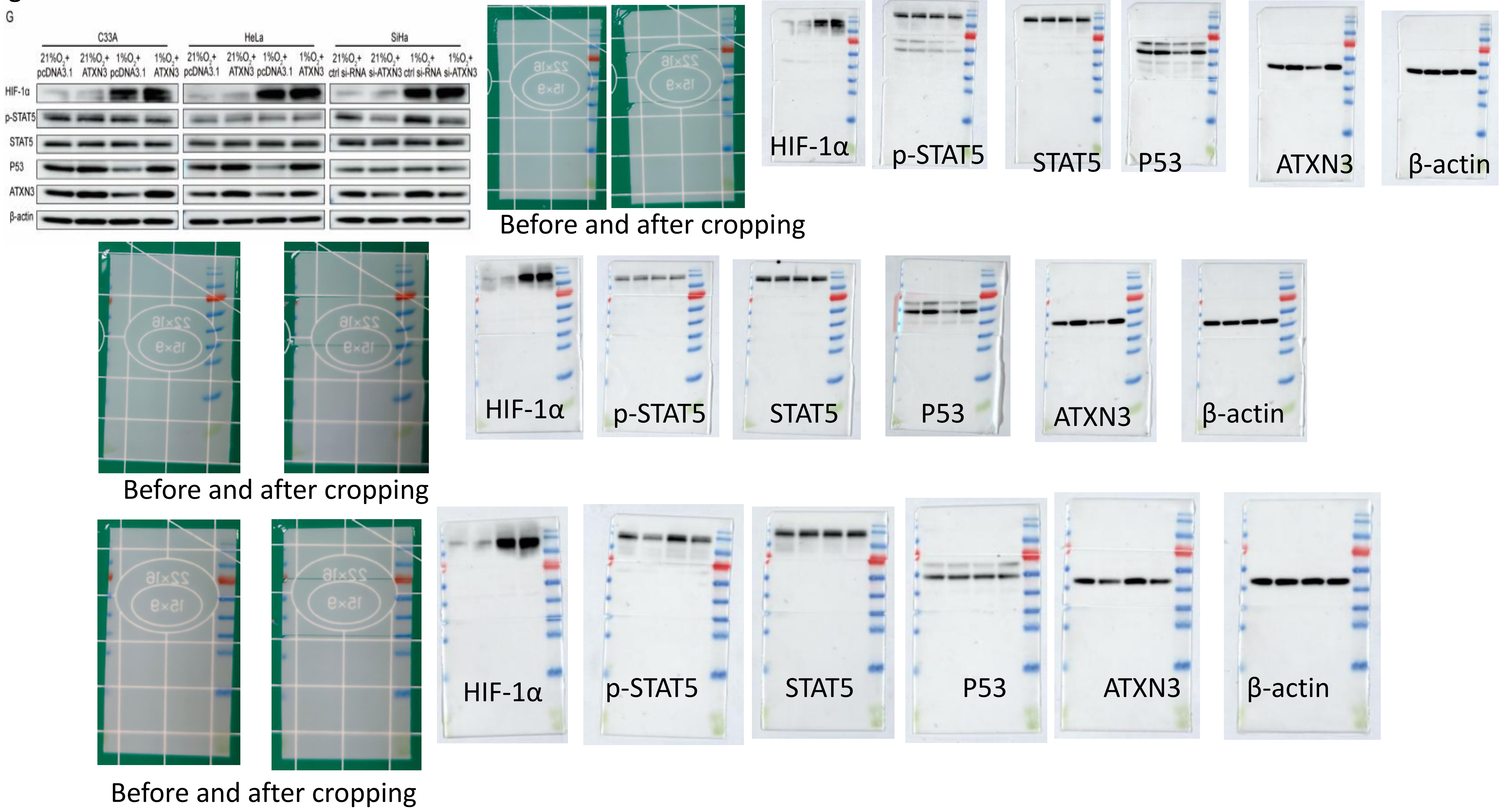
G
C33A
HeLa
SiHa
HIF-1α
p-STAT5
STAT5
P53
ATXN3
β-actin
Before and after cropping
HIF-1α
p-STAT5
STAT5
P53
ATXN3
β-actin
Before and after cropping
HIF-1α
p-STAT5
STAT5
P53
ATXN3
β-actin
Before and after cropping
HIF-1α
p-STAT5
STAT5
P53
ATXN3
β-actin
